# Mice carrying an analogous heterozygous dynamin 2 K562E mutation that causes neuropathy in humans develop predominant characteristics of a primary myopathy

**DOI:** 10.1093/hmg/ddaa034

**Published:** 2020-03-04

**Authors:** Jorge A Pereira, Joanne Gerber, Monica Ghidinelli, Daniel Gerber, Luigi Tortola, Andrea Ommer, Sven Bachofner, Francesco Santarella, Elisa Tinelli, Shuo Lin, Markus A Rüegg, Manfred Kopf, Klaus V Toyka, Ueli Suter

**Affiliations:** 1 Department of Biology, Institute of Molecular Health Sciences, Swiss Federal Institute of Technology, ETH Zurich, 8093 Zurich, Switzerland; 2 Biozentrum, University of Basel, Klingelbergstrasse 50/70, CH-4056 Basel, Switzerland; 3 Department of Neurology, University Hospital of Würzburg, University of Würzburg, 97080 Würzburg, Germany

## Abstract

Some mutations affecting dynamin 2 (*DNM2*) can cause dominantly inherited Charcot–Marie–Tooth (CMT) neuropathy. Here, we describe the analysis of mice carrying the DNM2 K562E mutation which has been associated with dominant-intermediate CMT type B (CMTDIB). Contrary to our expectations, heterozygous DNM2 K562E mutant mice did not develop definitive signs of an axonal or demyelinating neuropathy. Rather, we found a primary myopathy-like phenotype in these mice. A likely interpretation of these results is that the lack of a neuropathy in this mouse model has allowed the unmasking of a primary myopathy due to the DNM2 K562E mutation which might be overshadowed by the neuropathy in humans. Consequently, we hypothesize that a primary myopathy may also contribute to the disease mechanism in some CMTDIB patients. We propose that these findings should be considered in the evaluation of patients, the determination of the underlying disease processes and the development of tailored potential treatment strategies.

## Introduction

The peripheral nervous system (PNS) ensures the precise connection between the central nervous system (CNS) and various end organs including skeletal muscles. It shows specialized features to allow and guide efficient relay of neural information over potentially long distances (1). Schwann cells (SCs) enwrap peripheral axons of larger diameter with myelin, forming a multilayered compact membranous structure that secures fast saltatory conduction of action potentials in jawed vertebrates (2). The relation between adult SCs and peripheral axons is tuned to maximize conduction speed, which contrasts with the more complex and sometimes discontinuous myelination performed by oligodendrocytes in the CNS, where the system is more prone to adaptation and plasticity (3,4). PNS myelination is governed by continuous communication between SCs and neurons/axons, and disturbing either cell in this relationship can be detrimental to peripheral nerve function (5).

Charcot–Marie–Tooth (CMT) disease, also called hereditary motor and sensory neuropathies, is a genetically heterogeneous group of disorders caused by mutations that affect the functions of either SCs or axons primarily or both (6–9). Clinically, the different forms of CMT can be classified according to their mode of genetic transmission (9). Electrophysiological evaluations allow further refinements. Reduced nerve conduction velocities (NCVs) indicate demyelinating CMT, consistent with predominant myelin pathologies observed in nerve biopsies (CMT1). Normal or mildly reduced NCVs and reduced amplitudes of compound muscle action potentials (CMAPs) denote axonal CMT, coherent with a loss of myelinating large-calibre axons found in nerve biopsies (CMT2). An intermediate form of CMT (CMTI) has also been defined if the NCVs of different intrafamilial patients with the same mutation overlap with the NCVs of the two main groups (9). CMT patients typically display distal sensory loss and muscle weakness due to the peripheral nerve defects, followed by secondary muscle wasting that is associated with skeletal deformations including pes cavus, hammer toes and kyphoscoliosis (6,10).

**Figure 1:**
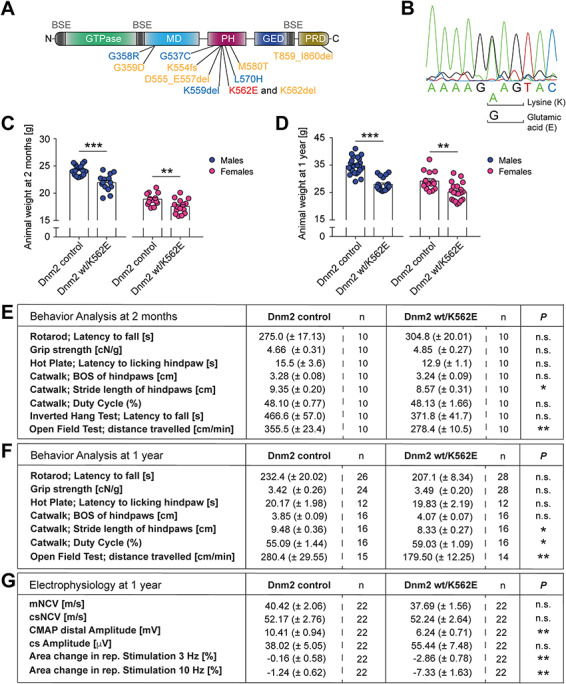
The Dnm2 wt/K562E mouse model. (**A**) Schematic representation of the various domains of DNM2. Key domains illustrated are the GTPase domain, the middle domain (MD), the pleckstrin homology domain (PH), the GTPase effector domain (GED) and the proline- and arginine-rich domain (PRD). The bundle signalling element (BSE) is represented in grey (68). Mutations found in patients with CMT2M (in blue; axonal neuropathy) and CMTDIB (in orange) are indicated. The DNM2 K562E mutation causes CMTDIB in humans and is highlighted in red. (**B**) Representative profile of sequencing of DNA extracted from Dnm2 wt/K562E mice confirming heterozygosity for adenine and guanine, which translate to lysine (K) and glutamic acid (E), respectively, at the correct genomic location. (**C**, **D**) Analysis of body weights of control and Dnm2 wt/K562E sex-specific mice at 2 months (C) and 1 year (D) of age. Both males and females have a slightly lower body mass in Dnm2 wt/K562E compared to their sex-specific controls at both ages. Bar heights, mean; error bars, SEM (females at 2 months, *n* = 13 control and *n* = 15 Dnm2 wt/K562E; females at 1 year, *n* = 15 control and *n* = 20 Dnm2 wt/K562E; males at 2 months, *n* = 17 control and *n* = 14 Dnm2 wt/K562E; males at 1 year, *n* = 24 control and *n* = 19 Dnm2 wt/K562E males). (**E**, **F**) Analysis of behaviour of 2-months-old (E) and 1-year-old (F) control and Dnm2 wt/K562E animals. General coordination and motor performance were assessed with rotarod and forepaw grip strength tests and, at 2 months of age, also by the inverted hang test. Sensory performance was examined with the hot plate test, gait analysis with the CatWalk device and total distance travelled by automated tracking in an open-field arena (over a 30-min period). Mutants displayed very mild changes in the gait analysis and travelled significantly shorter distances compared to controls in the open-field test at both 2 months and 1 year of age. Data refer to mean ± SEM (*n* refers to the number of mice/genotype examined in each test). (**G**) Electrophysiological assessment of 1-year-old control and Dnm2 wt/K562E mice. mNCV and csNCV are not significantly altered between control and Dnm2 wt/K562E animals. The CMAP amplitude, which includes the muscle response to the motor axon stimulation, is reduced in Dnm2 wt/K562E nerves. However, the nerve-specific compound sensory (cs) amplitude (depicts the sensory orthodromic and motor antidromic signal) is not significantly altered between both genotypes. Repetitive stimulation of the neuromuscular junctions produced at best a trend towards borderline synaptic fatigue at 3 and 10 Hz stimulation (*n* refers to the number of mice/genotype examined in each measurement). Two-tailed unpaired Student’s *t*-test. Significance was set at ^*^P < 0.05, ^**^P < 0.01 and ^***^P < 0.001.

*Dynamin 2* (*DNM2*) is one of the genes that, if affected by particular mutations (Fig. 1A), can cause either autosomal-dominant axonal CMT or dominant-intermediate CMT type B (CMTDIB). In this report, we concentrate on the particular CMTDIB-associated point-mutant DNM2 K562E (occasionally also called K558E, depending on the DNM2 isoform that was used as basis) (11–13). Patients harbouring this specific mutation usually experience disease onset in the first two decades of life and display gait impairments with various degrees of severity including an asymptomatic 18-year-old individual. Most DNM2 K562E patients present with weakness and muscle atrophy, more pronounced on distal compared to proximal portions of the lower limbs, combined with associated skeletal deformations including pes cavus (11). A subset of patients also develop cataracts and neutropenia. Electrophysiological analysis of DNM2 K562E patients revealed changes in both sensory and motor nerves. NCVs ranged from evidently decreased up to normal. Impaired CMAP amplitude was also detectable in some patients, with variable severity depending on the nerves analysed. Histological analysis of sural nerve biopsies from DNM2 K562E patients revealed loss of large-diameter axons, with the presence of regenerated myelinated axon clusters and focal hypermyelination (11,12). Acute axonal degeneration, segmental demyelination and remyelination with onion bulb formation were occasionally observed (11,12).

DNM2 is part of the dynamin family of large GTPases (14–16) and has been implicated in diverse cellular functions, including endocytosis, intracellular vesicular trafficking, regulation of cytoskeleton dynamics, mitosis and the biology of mitochondria (14,17–20). Not surprisingly, complete loss of DNM2 in mice is embryonic lethal, highlighting its key roles in development (21). Further studies aimed at elucidating the reliance of various cell types on DNM2 function revealed that, despite ubiquitous DNM2 expression, not all cells are fully dependent on this protein. Focusing on components of the neuromuscular circuit which are affected in CMT and autosomal-dominant centronuclear myopathy [CNM, a second disease associated with a distinct set of DNM2 mutations (22)], ablation of DNM2 specifically in mouse skeletal muscle resulted in muscle fibre atrophy and loss, altered neuromuscular junctions, degenerating nerve fibres, accumulation of lipid droplets and mitochondrial abnormalities (23). DNM2 loss exclusively in SCs revealed an essential function for correct developmental radial sorting of axons by SCs and in general SC survival, including in adult nerves (24). In contrast, some neural cells in the CNS appear to be less dependent on DNM2, possibly due to functional redundancy among different members of the dynamin family (24,25).

The studies indicated above were aimed at examining the consequences of DNM2 loss of function. With regard to CMT-associated DNM2 mutations, however, the mechanisms of action remain to be fully clarified (26). In biochemical experiments, DNM2 K562E displayed strong GTPase activity deficits (27), and in agreement with these results, DNM2 K562E displayed also impaired membrane fission activity (28). Furthermore, enforced expression of DNM2 K562E was unable to compensate for the lack of wild-type DNM2 in SCs using a myelination model of dissociated and cultured dorsal root ganglia (DRG) (29). Similarly, in a cell culture model not related to CMT, while fusion of osteoclasts was impaired upon simultaneous deletion of DNM1 and DNM2, expression of DNM2 K562E failed to restore normal fusion (30). Taken together, these data are consistent with some loss-of-function aspects of DNM2 K562E. Other experiments support, however, also a detrimental impact of the mutant protein. Overexpression of DNM2 K562E caused impaired clathrin-dependent endocytosis in fibroblast-like, motor neuron-like and SC cell lines (29,31,32). Furthermore, disturbed organization of the actin cytoskeleton was also observed in various cell lines upon DNM2 K562E overexpression (33).

Taken together, much of the current knowledge regarding the impact of DNM2 K562E is derived from overexpression of the mutant protein in various cell culture settings. However, the potential deleterious impact of elevated DNM2 K562E levels is limiting the interpretation of those experiments, in particular since increased expression of wild-type DNM2 can already have detrimental effects (26,34). Thus, since research models relying on wild-type or mutant DNM2 expression at non-physiological levels are at risk to produce some degree of artefacts (22), we reasoned that the analysis of a mouse model that carries the K562E mutation in the endogenous *Dnm2* gene, besides providing a valuable potential animal model for CMTDIB, is likely to yield additional knowledge about the impact of this mutation on the components of the neuromuscular circuit.

## Results

### The dominant CMTDIB-associated DNM2 K562E mutation does not cause an evident CMT-like neuropathy in wt/K562E mice

A mouse model carrying the Dnm2 K562E mutation (Fig. 1A) was generated by homologous recombination at the endogenous *Dnm2* locus. DNA sequencing confirmed the exchange of adenine by guanine in the mutant allele at the correct genomic location, yielding the desired heterozygous Dnm2 K562E mutant allele (Fig. 1B). Control and Dnm2 wt/K562E animals looked overall healthy, with no major alterations in their free-cage behaviour. Weight assessments revealed a slight reduction of the average body mass of Dnm2 wt/K562E mice, with substantial variability, in animals of both sexes at 2 months (Fig. 1C) and 1 year of age (Fig. 1D).

Since CMTDIB patients carrying the DNM2 K562E mutation show behavioural defects that affect both sensory and motor components, we evaluated Dnm2 wt/K562E mice with a collection of behavioural assays for sensory and motor performance. Assessments by the hot plate, rotarod and forepaw grip strength tests revealed no significantly impaired performance of Dnm2 wt/K562E mutants compared to controls in 2-month-old (Fig. 1E) and 1-year-old mice (Fig. 1F). Moreover, the inverted hang motor test, performed in 2-month-old animals, revealed no significant changes (Fig. 1E). Some minor gait alterations were detected in Dnm2 wt/K562E mice by the CatWalk analysis, mainly in the stride length, at both 2 months and 1 year of age (Fig. 1E and F). Notably, the slightly lower body mass of Dnm2 wt/K562E mice might have contributed to these subtle gait changes. When placed in an open-field arena and allowed to move freely for 30 min, Dnm2 wt/K562E mice moved consistently less than controls at both 2 months and 1 year of age (Fig. 1E and F). These findings, together with the observed subtle alterations in gait assessments, show that the Dnm2 K562E mutation mildly affects the locomotion of the mutant mice.

Electrophysiological evaluation of patients is a basic part of the diagnostic process for CMT, and CMTDIB patients with the DNM2 K562E mutation display various degrees of motor and sensory defects in this testing (11). Therefore, we performed electrophysiology measurements on Dnm2 wt/K562E and control mice. Assessing the PNS compartment, we observed no significant changes in the compound sensory (includes sensory orthodromic and motor antidromic) NCV (csNCV) and amplitude (cs Amplitude) (Fig. 1G). Examining the neural motor compartment specifically, we did not detect significant differences in motor NCV (mNCV) (Fig. 1G). Measuring both nerve and muscle performance, we found an evident decrease in the CMAP amplitude in Dnm2 wt/K562E mice (Fig. 1G). To evaluate whether a major fatigue effect of the neuromuscular junction is present in DNM2 wt/K562E mice, we performed repetitive stimulation analyses. These measurements revealed a slight, but significant, decrement of the muscle response at 3 and 10 Hz (Fig. 1G). However, the decrements observed were subtle and unlikely to reflect a physiologically relevant impairment of neuromuscular transmission. In summary, the functional electrophysiology analyses revealed no major damage of the neural component in Dnm2 wt/K562E animals. When muscles are included in the assessment, however, deficits were detectable in Dnm2 wt/K562E mice compared to controls. These alterations may contribute to the changes in the gait and the open-field mobility of mutants.

**Figure 2:**
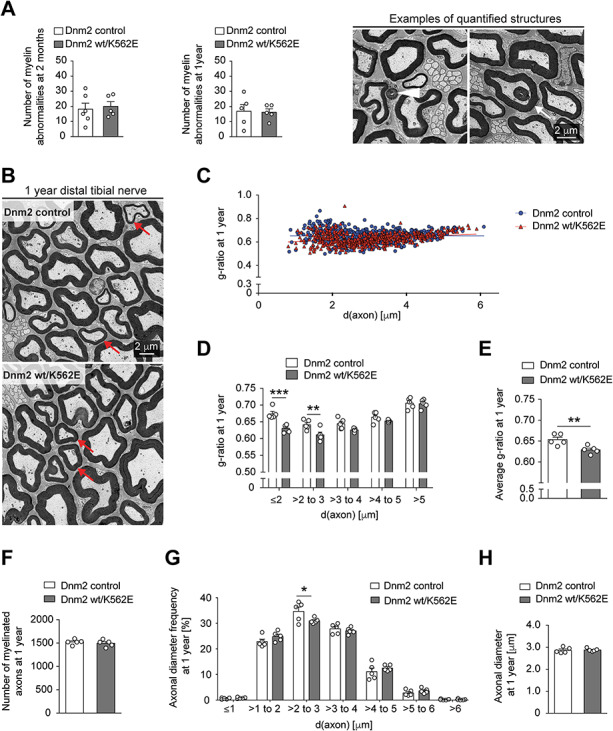
The Dnm2 wt/K562E mouse displays no overt histological signs of a neuropathy. (**A**) Evaluation of myelin abnormalities including outfoldings (white arrowhead) and infoldings (white arrow) throughout entire cross sections of distal tibial nerves in EM panoramas reveals no significant differences between control and Dnm2 wt/K562E nerves at 2 months and 1 year of age. Bar heights, mean; error bars, SEM (*n* = 5 mice for Dnm2 wt/K562E at both time points and for controls at 1 year, *n* = 6 mice for controls at 2 months). Example images, derived from 2-month-old Dnm2 wt/K562E mutants, are used to show quantified features. (**B**) Exemplary EM picture of distal tibial nerve cross sections of control and Dnm2 wt/K562E animals at 1 year of age, highlighting axons of similar, relatively small, diameter between both genotypes (red arrows) which display thicker myelin in the Dnm2 wt/K562E sample. Scale bar: 2 μm, refers to the whole panel. (**C**) Scatter plot of g-ratio versus axonal diameter derived from the analysis of EM micrographs from 1-year-old distal tibial nerve. Three random areas were quantified within the cross-section panorama, and all myelinated axons (excluding those showing myelin abnormalities) located within these areas were measured. Every datapoint represents one axon (*n* = 5 mice/genotype; at least 100 axons from each animal were quantified). (**D**, **E**) Binned g-ratio distribution per axonal diameter (D) and average g-ratio (E) of 1-year-old distal tibial nerves (same nerves analysed as depicted in C). Dnm2 wt/K562E mice show a slightly lower g-ratio, especially on smaller-calibre axons, indicative of thicker myelin compared to control animals. Bar heights, mean; error bars, SEM (*n* = 5 mice/genotype). (**F**–**H**) Number of myelinated axons (F), frequency distribution of axonal diameter bins (G) and average axonal diameter (H). All myelinated axons (F) and myelinated axons without myelin abnormalities (G, H) were measured on the entire cross section of distal tibial nerve EM panoramas at 1 year of age. No detectable systematic shrinking of Dnm2 wt/K562E axons relative to controls was found. Bar heights, mean; error bars, SEM (*n* = 5 mice/genotype). Two-way ANOVA with Sidak’s multiple comparisons test (D, G) and two-tailed unpaired Student’s *t*-test (A, E, F, H). Significance was set at ^*^P < 0.05, ^**^P < 0.01 and ^***^P < 0.001.

To evaluate the phenotype of Dnm2 wt/K562E mice further, we carried out a detailed histological analysis of peripheral nerves, as occasionally performed also in patient biopsies for refined diagnostic purposes. CMT tends to be slowly progressive over time and is usually more evident in the lower limbs (10). Thus, we focused our histological analysis on distal tibial nerves of mice aged 1 year and compared these to young 2-month-old adults. First, we searched for a potential myelination phenotype since increased frequencies of myelin abnormalities, including myelin infoldings, myelin outfoldings and focal hypermyelination (also known as tomacula), are characteristic of certain forms of dysmyelinating/demyelinating CMT, and some of these features have been reported in DNM2 wt/K562E (CMTDIB) human biopsies (11). Evaluations of entire nerve cross sections by electron microscopy (EM) revealed minor morphological abnormalities in Dnm2 wt/K562E nerves and controls, but no significant differences were found (Fig. 2A). Furthermore, we found no major signs of demyelination or remyelination. Analyses of g-ratio (i.e. the ratio of the axonal diameter divided by the fibre diameter) revealed slightly lower average values in 1-year-old Dnm2 wt/K562E nerves (Fig. 2E) compared to controls, indicating marginally thicker myelin sheaths in mutants, especially surrounding small calibre axons (Fig. 2B–D). Myelin periodicity was not detectably altered between aldehyde-fixed nerves of Dnm2 wt/K562E animals and controls (Supplementary Material, Fig. S1). Analyses of 2-month-old mice yielded no significant changes in g-ratio (Supplementary Material, Fig. S2A–D) between Dnm2 wt/K562E and control animals. Taken together and consistent with the electrophysiology data, these observations indicate that Dnm2 wt/K562E animals do not develop typical features of a dysmyelinating/demyelinating neuropathy even up to 1 year of age.

Since the DNM2 K562E mutation in humans leads to an intermediate neuropathy with loss of large-diameter axons, a characteristic feature of axonal CMT too, we next assessed whether Dnm2 K562E mice develop signs of an axonal phenotype. Such an analysis was further warranted since the observed reduced CMAP amplitude in the mixed motor nerve and muscle readout of Dnm2 K562E mice is consistent with an axonal form of CMT. We found no significant changes in the number of myelinated axons comparing Dnm2 K562E mouse nerves to controls (Fig. 2F and Supplementary Material, Fig. S2E). Furthermore, analogous to previous reports (35,36), we evaluated the sizes of all myelinated axons (without myelin abnormalities) covering the cross-section area of distal tibial nerves of 1-year-old (Fig. 2G and H) and 2-month-old mice (Supplementary Material, Fig. S2F and G). At both ages, we found no evidence for loss of large-diameter axons or for a major reduction in axonal diameters in Dnm2 wt/K562E nerves relative to controls. Taken together, our data do not indicate a prominent axonal pathology in Dnm2 wt/K562E animals.

### In SCs, the Dnm2 K562E allele alone is able to fulfil the crucial function of DNM2 in radial axonal sorting

Given the apparent lack of a major effect of the mutant *Dnm2* K562E allele on the function of Dnm2 wt/K562E adult nerves, we sought to examine the nature of the impact of this mutation in the neural compartment under physiological conditions further. We have previously found that Dnm2 in SCs is essential for radial axonal sorting, a critical event in nerve development that is required to separate individual large-calibre axons in a 1:1 relation with SCs as a prerequisite for PNS myelination (24). Thus, we asked whether SCs containing the *Dnm2* K562E allele alone, i.e. without wild-type Dnm2 expression, are able to drive the radial sorting event accurately. To address this question, we bred Dnm2 wt/K562E mice with mice expressing Cre recombinase under the *Mpz* promoter (P0Cre) (37) and mice carrying conditional null *Dnm2* floxed (fl) alleles (24). The resulting offspring yielded P0Cre Dnm2 fl/K562E mice that contain the *Dnm2* K562E allele but lack wild-type DNM2 in SCs. Detailed analyses of these mice at postnatal day 5, a time point when axonal sorting and myelination onset defects are prominent in sciatic nerves (SNs) of mice lacking DNM2 in SCs (24), revealed no detectable morphological changes in tibial nerves (Supplementary Material, Fig. S3A–E) or SNs (Supplementary Material, Fig. S3F–J) if compared to controls and also Dnm2 K562E mice. Thus, the presence of the *Dnm2* K562E allele alone, without a Dnm2 wild-type allele in SCs, is sufficient to allow successful radial sorting and correct onset of myelination by SCs. This stands in contrast to the strong defects observed in P0Cre Dnm2 fl/fl nerves that are devoid of DNM2 in SCs all together (24). The most straightforward interpretation of these results is that the Dnm2 K562E protein has retained the required functionality to guide early PNS development accurately. Alternatively, the presence of the *Dnm2* K562E allele may be able to induce actively adequate compensatory mechanisms.

Overexpression of the mutant Dnm2 K562E protein has been shown to decrease CME in various cell types, including SCs (29,31). To test whether the *Dnm2* K562E allele, either alone (P0Cre Dnm2 fl/K562E) or combined with the wild-type allele (P0Cre Dnm2 wt/K562E), is associated with impaired CME in SCs in a more physiological setting, we performed uptake assays of fluorescently labelled transferrin on primary cultured SCs isolated from neonatal nerves of our transgenic mice. The *Rosa26-loxPstoploxP-YFP* allele was also included to mark SCs in these experiments, in this way allowing the application of a FACS-based assay that has been used previously to demonstrate the strongly reduced CME of SCs that lack DNM2 completely (24). We did not find significantly altered levels of internalized labelled transferrin in SCs derived from P0Cre Dnm2 fl/K562E or P0Cre Dnm2 wt/K562E compared to controls (Supplementary Material, Fig. S3K). Thus, we conclude that the observed normal levels of CME in those mutant SCs may contribute to the apparently accurate early PNS development in the corresponding mutant animals.

### Inactivation of the remaining *Dnm2* wild-type allele in SCs carrying the *Dnm2* K562E allele leads to the fast development of a demyelinating neuropathy-like phenotype

**Figure 3:**
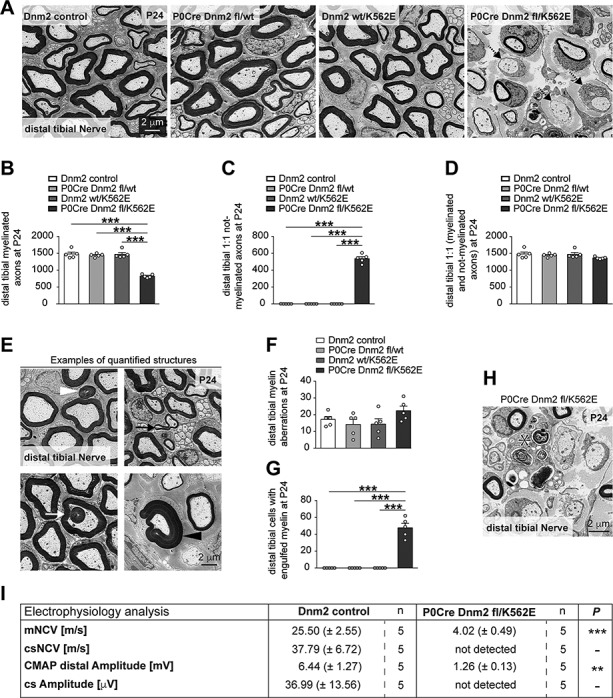
Inactivation of the wild-type *Dnm2* allele from SCs of mice carrying the Dnm2 K562E allele leads to rapid development of a neuropathy-like phenotype. (**A**) Exemplary EM micrographs of cross sections of distal tibial nerves of control, P0Cre Dnm2 fl/wt, Dnm2 wt/K562E and P0Cre Dnm2 fl/K562E at P24. Dashed arrows highlight myelin-competent axons (>1 μm diameter) without myelin. Scale bar: 2 μm, refers to whole panel. (**B**–**D**) Quantification of myelinated axons (B) and 1:1 not-myelinated SC-axon profiles (C, axons >1 μm diameter) reveals abundant axons deprived of myelin in P24 P0Cre Dnm2 fl/K562E, but not in the other genotypes. The total number of sorted axons (D, includes myelinated and 1:1 not-myelinated axons >1 μm diameter) shows no significant loss of axons in any of the analysed genotypes compared to controls. Whole cross-section EM panoramas (P24) were quantified. Bar heights, mean; error bars, SEM (*n* = 5 mice/genotype). (**E**, **F**) Evaluation of myelin abnormalities including outfoldings (white arrowhead), infoldings (white arrow), tomacula (black arrowhead) and comma-shaped structures (black arrow) throughout the entire cross section of P24 distal tibial nerves in EM panoramas shows a low total number of abnormalities, which are not significantly different between control, P0Cre Dnm2 fl/wt, Dnm2 wt/K562E and P0Cre Dnm2 fl/K562E mice. The figure with exemplary tomacula was taken from P0Cre Dnm2 fl/K562E mice, whereas the other exemplary features were taken from Dnm2 wt/K562E mice. Scale bar: 2 μm, refers to whole panel. Bar heights, mean; error bars, SEM (*n* = 5 mice/genotype). (**G**, **H**) Quantification throughout the entire cross section of P24 distal tibial nerves in EM panoramas (G) of cells containing engulfed myelin (H, asterisk) reveals a selective enrichment in P0Cre Dnm2 fl/K562E, but not in the other genotypes, supporting that demyelination is likely to occur and contributes to the not-myelinated axons quantified in (C). Scale bar: 2 μm. Bar heights, mean; error bars, SEM (*n* = 5 mice/genotype). (**I**) Electrophysiology analysis in control and P0Cre Dnm2 fl/K562E mice between 3 and 4 weeks of age. The mNCV is severely decreased in mutant animals, and the corresponding CMAP amplitude is also lower in mutants compared to controls under supramaximal stimulation conditions. The csNCV and cs Amplitude could not be detected in mutant animals under the chosen study conditions, while a robust response was detected in all control animals evaluated (*n* refers to the number of mice/genotype and is indicated in the figure for every measurement). One-way ANOVA with Tukey’s multiple comparisons test (B–D, F, G) and two-tailed unpaired Student’s *t*-test (I). Significance was set at ^**^P < 0.01 and ^***^P < 0.001.

We were somewhat surprised by the finding that early peripheral nerve maturation (i.e. radial sorting and onset of myelination) can be maintained by the sole presence of the *Dnm2* K562E allele in SCs of P0Cre Dnm2 fl/K562E mice. Thus, we wondered whether also all other functions of SC-DNM2 that are required to maintain myelinated nerves can be preserved by the Dnm2 K562E allele, keeping in mind that even induced loss of DNM2 in SCs of adult mice causes a strong (transient) neuropathy-like phenotype (24). To approach this question, we analysed P0Cre Dnm2 fl/K562E nerves compared to controls at P24. We found a strong reduction of myelinated axons in P0Cre Dnm2 fl/K562E nerves (Fig. 3A and B), paired with a proportional increase in the number of sorted myelin-competent axons (i.e. >1 μm in diameter) that were not enwrapped by myelin (Fig. 3A and C). To assess potential axonal degeneration, we compared the number of sorted axons (i.e. the sum of myelinated and not-myelinated 1:1 myelin-competent profiles) but did not detect significant differences (Fig. 3D). Also, the numbers of structural myelin aberrations were not changed (Fig. 3E and F), but cells with engulfed myelin inclusions, indicative of ongoing demyelination, were commonly found in P0Cre Dnm2 fl/K562E nerves but not in controls (Fig. 3G and H). Dnm2 fl/wt nerves were also included in these analyses to examine whether the presence of a single *Dnm2* wild-type allele in SCs can lead to neuropathic features, but we found no significant changes compared to controls (Fig. 3A–G). Furthermore, a parallel analysis of Dnm2 K562E nerves at P24 revealed no alterations (Fig. 3A–G), in agreement with the previous analyses at 2 months of age. We evaluated the functional consequences of the pathology observed in P0Cre Dnm2 fl/K562E mice also by electrophysiology (Fig. 3I). These studies revealed drastically reduced mNCV, together with decreased CMAP amplitude compared to controls. The analysis of csNCV and cs Amplitude showed the expected robust performance in controls, while the neuroelectric signal was not detectable in P0Cre Dnm2 fl/K562E mice. Taken together, P0Cre Dnm2 fl/K562E mice show multiple hallmarks of a rather severe demyelinating neuropathy. In a more mechanism-focused summary, our results indicate that the Dnm2 K562E allele in SCs is not capable of maintaining the myelinated state of SCs, with the corresponding pathological and electrophysiological features ensuing. Combined with the results in early development, the data indicate that the *Dnm2* K562E allele in SCs alone is capable to sustain some aspects of normal peripheral nerve biology, but not all of them. The elucidation of the precise mechanisms behind these tantalizing findings will be a fascinating topic for future analyses.

**Figure 4:**
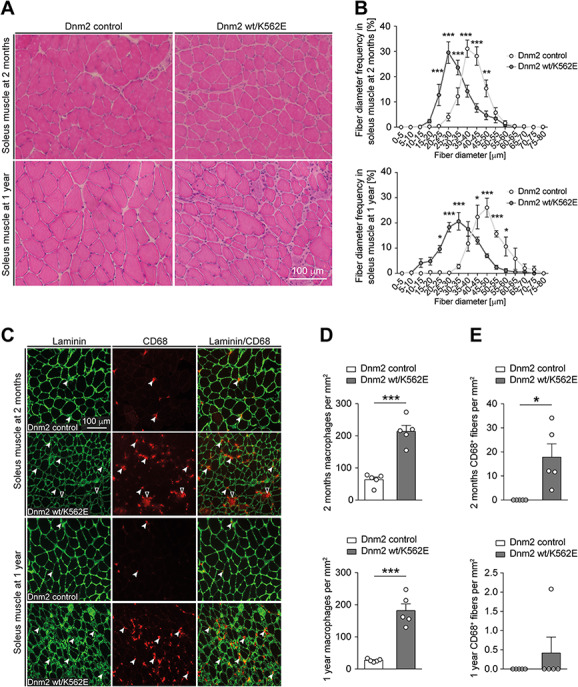
The Dnm2 wt/K562E mouse develops an evident myopathy. (**A)** Exemplary H&E-stained cryosections of soleus muscles derived from control and Dnm2 wt/K562E mice at 2 months and 1 year of age. Scale bar: 100 μm, refers to whole panel. (**B**) Quantification of myofibre diameter frequencies reveals a shift towards smaller-calibre fibres in Dnm2 wt/K562E soleus muscles at both 2 months and 1 year of age. Datapoints, mean; error bars, SEM (*n* = 5 mice/genotype, images from three sections quantified and averaged per mouse). (**C**) Exemplary soleus muscle cryosections from control and Dnm2 wt/K562E probed with antibodies targeting laminin or CD68 at both 2 months and 1 year of age. Exemplary CD68-positive macrophages in between myofibres (white arrowheads) and from within the myofibre basal lamina (black arrowheads with white lining) are highlighted. Scale bar: 100 μm, refers to whole panel. (**D**, **E**) Quantification of CD68-positive macrophages in between myofibres (D) reveals a significant enrichment in Dnm2 wt/K562E mice at both 2 months and 1 year, and quantification of CD68-positive macrophages from within myofibre basal lamina (E) reveals a significant increase in Dnm2 wt/K562E soleus muscles at 2 months. Bar heights, mean; error bars, SEM (*n* = 5 mice/genotype, images of at least three sections quantified and averaged per mouse). Two-way ANOVA with Sidak’s multiple comparisons test (B), two-tailed unpaired Student’s *t*-test (D) and one-sample *t*-test (E). Significance was set at ^*^P < 0.05, ^**^P < 0.01 and ^***^P < 0.001.

### DNM2 wt/K562E mice develop an enduring primary myopathy

The electrophysiological evaluation performed in Dnm2 wt/K562E nerves at 1 year of age revealed a robust decrease of the CMAP response (Fig. 1G), which is consistent with problems either in motor neurons or in the muscle itself (38). Considering that we detected no impairments on nerve-specific electrophysiology parameters (Fig. 1G) and that we failed to find morphological evidence for a neuropathy in Dnm2 wt/K562E mice at 2 months and 1 year of age (Fig. 2 and Supplementary Material, Fig. S1), we reasoned that the CMAP defect may derive from a muscle phenotype in Dnm2 wt/K562E animals. Thus, we evaluated the soleus muscles of 2-month-old and 1-year-old Dnm2 wt/K562E and control mice. Evaluations of the distributions of fibre diameter frequencies in haematoxylin-eosin (H&E)-stained soleus muscle cross sections revealed a shift towards smaller-calibre fibres in Dnm2 wt/K562E mice compared to control mice at both ages (Fig. 4A and B), suggestive of a myopathy. Myofibre damage is usually coupled to macrophage accumulation to ensure debris clearance (39,40). To assess the abundance of macrophages, we performed immunohistochemistry of soleus muscle cryosections using antibodies recognizing CD68, combined with myofibre outlines labelled with laminin antibodies. The numbers of macrophages in between myofibres were significantly elevated in Dnm2 wt/K562E animals compared to controls at both ages examined (Fig. 4C and D). Macrophages are also known to scavenge damaged myofibres from within the basal lamina. Consistently, we detected a variable but significantly increased presence of these features in Dnm2 wt/K562E compared to control soleus muscles in 2-month-old mice (Fig. 4C and E). Damage of myofibres during a myopathy may further be associated with increased deposition of ECM/fibrotic tissue. We evaluated ECM distribution in the soleus muscles using Masson’s trichrome staining and observed a more widespread staining in Dnm2 wt/K562E compared to control sections at 2 months and 1 year of age (Supplementary Material, Fig. S4A). Fibre regeneration from satellite cells is common after muscle damage. The newly maturing myofibres express specific types of myosin subunits that are normally absent from mature fibres. Immunohistochemistry analysis of soleus muscles from 2-month-old animals, using antibodies targeting myosin subunits specific to embryonic or regenerating fibres, revealed significantly increased albeit low and variable levels of these aberrant structures in Dnm2 wt/K562E soleus sections compared to controls (Supplementary Material, Fig. S4B and C). In accordance with a myopathy and reduced fibre size, the weight of soleus muscles (including the calcaneal tendon, used for handling the tissue) was significantly lower in Dnm2 wt/K562E male and females mice compared to respective sex controls at 1 year of age and also already significantly reduced in 2-month-old Dnm2 wt/K562E males (Supplementary Material, Fig. S4D). To assess whether the lasting myopathy is related to an increase in centronucleated fibres, a cardinal feature in CNM patients with a distinct set of DNM2 mutations, we quantified the number of fibres with central nuclei in soleus muscles of 1-year-old Dnm2 wt/K562E and control mice but detected no significant changes (Supplementary Material, Fig. S4E). Taken together, our results indicate a mild but definitive and enduring myopathy-like phenotype in Dnm2 wt/K562E mice, which develops in the absence of a detectable neuropathy.

Since the DNM2 K562E mutation causes a neuropathy in patients, we set out to evaluate whether this mutation leads to widespread denervation of muscles that could majorly contribute to muscle wasting. To explore whether evident signs of denervation are detected in the soleus muscles of Dnm2 wt/K562E mice, we stained the presynaptic terminals with synaptophysin antibodies, and the postsynaptic neuromuscular junctions with α-bungarotoxin, on whole-mount preparations of soleus myofibres. This analysis revealed a marginal decrease of fully innervated endplates at both 2 months and 1 year of age and a slight increase of partially denervated endplates in 1-year-old Dnm2 wt/K562E mice. However, we did not detect a significant increase of fully denervated fibres at either time point (Supplementary Material, Fig. S5A–D). Another sign of denervation and re-innervation would be fibre type-specific regrouping in the muscles. To assess fibre-type grouping, we used antibodies specifically recognizing the myosin heavy chains of fibre type I, fibre type IIA and fibre type IIB. Immunohistochemistry on soleus muscle sections revealed the typical random checkerboard pattern of fibre-type distribution in both Dnm2 wt/K562E and control mice (Supplementary Material, Fig. S6A). Quantification of fibre-type abundance revealed a very small, but statistically significant, increase of fibre type II and decrease of fibre type I at both 2 months and 1 year of age (Supplementary Material, Fig. S6B). Interpreting these data, we favour that the small differences in fibre-type abundance, and also the small reduction of fully innervated endplates, are minimal changes that are probably the result of the persistent myopathy and are not likely to indicate physiologically relevant primary denervation.

**Figure 5:**
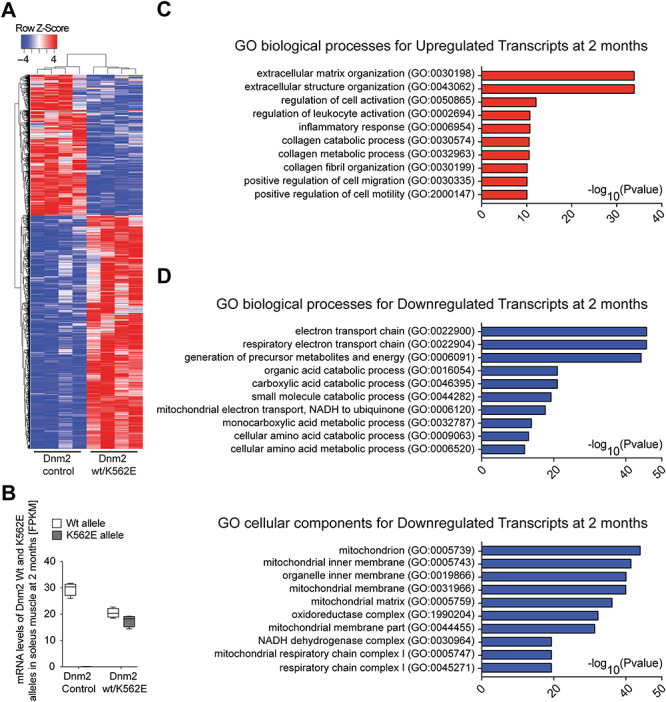
Transcriptome analyses of Dnm2 wt/K562E soleus muscle reveals differential regulation of transcripts consistent with a myopathy. (**A**) Heatmap of differentially regulated transcripts (FDR < 0.05 and log2 fold change at least ±0.58, approximately ±1.5-fold in linear scale) between control and Dnm2 wt/K562E 2-month-old soleus muscle RNA extracts. Each column represents one mouse. Cluster analysis reveals that control and mutant mice cluster properly with other mice of the same genotype (*n* = 4 mice/genotype). (**B**) Box and whiskers plot depicting the number of transcripts (FPKM) expressed by the Dnm2 wild-type and the Dnm2 wt/K562E allele, both in control and Dnm2 wt/K562E soleus muscle at 2 months of age. Each allele produces a similar number of transcripts in Dnm2 wt/K562E soleus samples. Error bars: minimum to maximum range (*n* = 4 mice/genotype). (**C**) GO of biological process analysis of upregulated transcripts from Dnm2 wt/K562E versus control soleus (FDR < 0.05 and log2 fold change ≥0.58) reveals that inflammation and ECM organization are among the most significantly represented categories in the dataset (*n* = 4 mice/genotype). (**D**) GO of biological processes and GO of cellular components analysis of downregulated transcripts from Dnm2 wt/K562E versus control soleus (FDR < 0.05 and log2 fold change ≤−0.58) reveals that metabolic pathways associated with mitochondrial function, and mitochondria as an organelle, dominate the most significantly represented categories in the dataset (*n* = 4 mice/genotype).

Our morphological assessment points to a primary myopathy-like phenotype in Dnm2 wt/K562E mice. To evaluate the transcriptional impact of the Dnm2 K562E mutation in myofibres, we extracted RNA from soleus muscles of 2-month-old Dnm2 wt/K562E and control animals, followed by RNA sequencing. The significantly differentially regulated transcripts were shortlisted based on FDR < 0.05 and log2 fold change ±0.58 (approximately equivalent to 1.5-fold up- or downregulation on a linear scale). These selection criteria resulted in 1934 upregulated and 1157 downregulated transcripts. Differential-cluster analysis revealed a close agglomeration of the individual control animals in one group and the individual mutant animals in a second group, with a coherent heatmap pattern of up- and downregulated transcripts (Fig. 5A). Specific alignments of reads containing the Dnm2 K562E point mutation and corresponding wild-type transcripts confirmed the absence of the Dnm2 K562E mutation in controls and showed a similar abundance of Dnm2 wild-type and Dnm2 K562E transcripts in the soleus muscle of Dnm2 wt/K562E mice (Fig. 5B), indicating that there is no allelic dominance of expression in Dnm2 wt/K562E muscles. To assess whether the differentially regulated transcripts can expand the knowledge about the cellular and molecular phenotype of Dnm2 wt/K562E muscles, or even suggest potential causes of the myopathy, we performed Gene Ontology (GO) analysis using EnrichR. Regarding upregulated transcripts in Dnm2 wt/K562E mice, GO of biological processes highlighted extracellular matrix organization and also inflammation as dominant topics within the 10 most significantly upregulated categories (Fig. 5C). The increase of extracellular matrix-related RNAs is likely related to the accumulation of ECM in Dnm2 wt/K562E muscle detected in Masson’s trichrome staining (Supplementary Material, Fig. S4A), and the increase of inflammation-associated transcripts is also coherent with the increased number of macrophages in Dnm2 wt/K562E muscles (Fig. 4C). In the GO of biological process analysis for downregulated transcripts, mitochondrial oxidative phosphorylation and energy-related metabolism dominated within the 10 most significantly downregulated categories (Fig. 5D, upper part). These alterations of prominent mitochondria-related functions prompted us to extend the GO analysis to cellular components. Coherently, the 10 most significantly downregulated categories in GO cellular components analysis were related to mitochondria and the oxidoreductase respiratory chain (Fig. 5D, lower part). As an orthogonal informatic approach to evaluate the globally downregulated transcripts, we loaded our dataset of upregulated and downregulated transcripts also into IPA Ingenuity software. The ‘canonical pathway’ analysis of Ingenuity revealed (a) oxidative phosphorylation and (b) mitochondrial dysfunction as the most significant categories associated with these transcripts (Supplementary Material, Fig. S7). Heatmap representation of the transcripts associated with these two IPA categories in our dataset confirmed that the majority of these transcripts are downregulated in Dnm2 wt/K562E soleus muscles (Supplementary Material, Fig. S7). Taken together, the RNA sequencing analysis highlights a major transcriptome impact of the Dnm2 K562E mutation. The Gene Ontology (GO) categories of upregulated transcripts corroborate the ECM deposition and increased number of macrophages in Dnm2 wt/K562E muscles as described in our other experiments, while the analysis of downregulated transcripts revealed a surprising dominance of mitochondria-associated oxidative phosphorylation and mitochondrial dysfunction.

### The myopathy in Dnm2 wt/K562E mice extends beyond the soleus muscle

**Figure 6:**
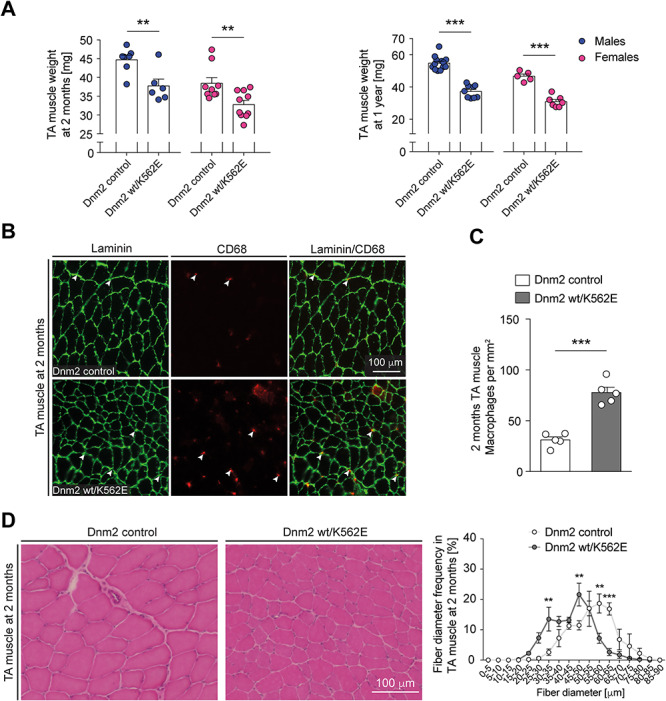
The myopathic features in Dnm2 wt/K562E mice are not restricted to the soleus muscle. (**A**) Weight analysis of the TA of control and Dnm2 wt/K562E sex-specific mice at 2 months and 1 year of age. The muscles have a lower mass in Dnm2 wt/K562E males and females compared to sex-specific controls at both ages. Bar heights, mean; error bars, SEM (females at 2 months, *n* = 9 control and *n* = 10 Dnm2 wt/K562E; females at 1 year, *n* = 5 control and *n* = 7 Dnm2 wt/K562E; males at 2 months, *n* = 7 control and *n* = 6 Dnm2 wt/K562E; males at 1 year, *n* = 13 control and *n* = 9 Dnm2 wt/K562E). (**B**) Exemplary cryosections of TAs derived from 2-month-old control and Dnm2 wt/K562E mice, probed with antibodies against laminin or CD68. Exemplary CD68-positive macrophages (white arrowheads) are highlighted. Scale bar: 100 μm, refers to whole panel. (**C**) Quantification of CD68-positive macrophages in between myofibres reveals a significant enrichment in Dnm2 wt/K562E TAs at 2 months. Bar heights, mean; error bars, SEM (*n* = 5 mice/genotype, images of three sections quantified and averaged per mouse). (**D**) (Left) Exemplary H&E-stained TA cryosections derived from control and Dnm2 wt/K562E mice at 2 months. Scale bar: 100 μm, refers to whole panel. (D) (Right) Quantification of myofibre diameter frequencies shows a shift towards smaller-calibre myofibers in Dnm2 wt/K562E TA at 2 months. Datapoints, mean; error bars, SEM (*n* = 4 mice/genotype, images from three sections quantified and averaged per mouse). Two-tailed unpaired Student’s *t*-test (A, C) and two-way ANOVA with Sidak’s multiple comparisons test (D). Significance was set at ^**^P < 0.01 and ^***^P < 0.001.

The observation of myopathic features in the soleus muscles of Dnm2 wt/K562E mice prompted the question of whether the myopathy is restricted to this muscle. To assess this issue, we evaluated key features also in the tibialis anterior muscle (TA) of 2-month-old mice, matching a stage at which the soleus is evidently affected in Dnm2 wt/K562E mice. The analysis of muscle weights revealed a significant reduction in both male and female Dnm2 wt/K562E mice compared to controls, with some variability (Fig. 6A, left) reaching greater uniformity at 1 year of age (Fig. 6A, right). Assessment of CD68-positive cells revealed a consistent increase in the number of macrophages populating Dnm2 wt/K562E muscles compared to controls (Fig. 6B and C). Finally, evaluation of fibre size in H&E-stained cryosections demonstrates a shift in fibre diameter frequency distributions towards smaller-calibre fibres in Dnm2 wt/K562E mice (Fig. 6D). Taken together, these observations in the mutant TA recapitulate key myopathic features of the mutant soleus muscle and confirm that the myopathy in Dnm2 wt/K562E mice affects multiple skeletal muscles.

**Figure 7:**
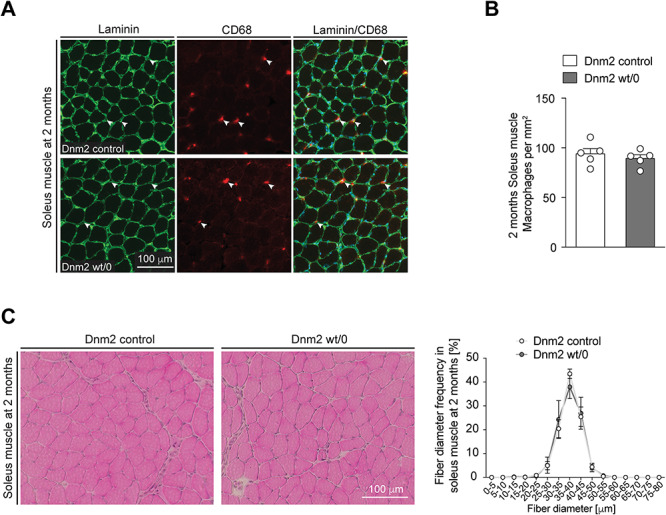
DNM2 haploinsufficiency is not the likely underlying mechanism of the myopathy in Dnm2 K562E mice. (**A**) Exemplary cryosections of soleus muscles derived from 2-month-old control and Dnm2 wt/0 mice probed with antibodies targeting laminin or CD68. Exemplary CD68-positive macrophages (white arrowheads) are highlighted. Scale bar: 100 μm, refers to whole panel. (**B**) Quantification of CD68-positive macrophages in between myofibres reveals no significant changes between control and Dnm2 wt/0 muscles at 2 months. Bar heights, mean; error bars, SEM (*n* = 5 mice/genotype, images from two to three sections quantified and averaged per mouse). (**C**) (Left) Exemplary H&E-stained soleus muscle cryosections derived from control and Dnm2 wt/0 mice at 2 months. Scale bar: 100 μm, refers to whole panel. (C) (Right) Quantification of myofibre diameter frequency reveals no detectable difference between both genotypes on soleus muscle sections at 2 months. Datapoints, mean; error bars, SEM (*n* = 5 mice/genotype, images from three sections quantified and averaged per mouse). Two-tailed unpaired Student’s *t*-test (B) and two-way ANOVA with Sidak’s multiple comparisons test (C).

### DNM2 haploinsufficiency is not the underlying mechanism of the myopathy in Dnm2 K562E mice

Our analysis thus far had revealed a mild but definitive myopathy in Dnm2 wt/K562E mice. With regard to the human disease caused by the analogous genetic mutation, we asked whether the presence of a single wild-type *Dnm2* allele alone, i.e. haploinsufficiency without a necessary contribution by the *Dnm2* K562E allele, is sufficient to cause a myopathy, at least in mice. To address this question, we used a previously generated *Dnm2* null allele to obtain Dnm2 wt/0 mice (29). Evaluations of the soleus muscles of such mice revealed no significant changes either in the number of CD68-positive macrophages (Fig. 7A and B) or in the frequency distribution of fibre diameters in H&E-stained cryosections (Fig. 7C) compared to controls at 2 months of age. Since these parameters were altered in Dnm2 wt/K562E mice, the data indicate that a single gene copy of *Dnm2* is sufficient to preserve soleus muscle health in mice. Consequently, haploinsufficiency cannot be the only reason for the myopathic features present in Dnm2 wt/K562E mice.

### Dnm2 wt/K562E mice do not develop evident signs of neutropenia

**Figure 8:**
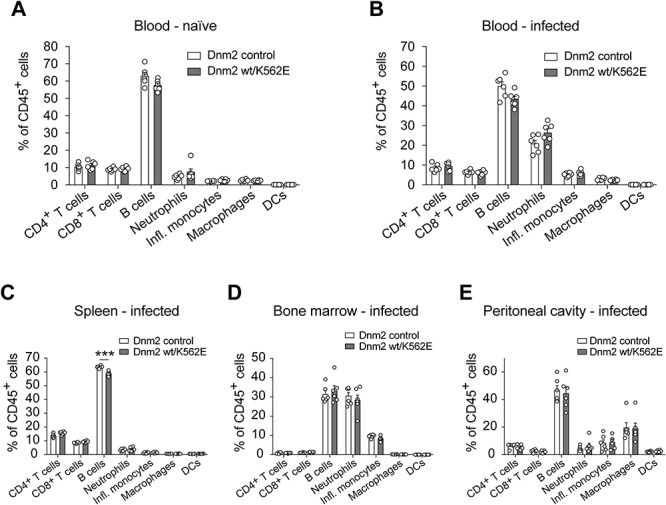
Dnm2 wt/K562E mice do not reproduce obvious signs of neutropenia as reported in some human patients carrying the same mutation. (**A**–**E**) Analysis of the relative abundance of immune cells (CD4^+^ T cells, CD8^+^ T cells, B cells, neutrophils, inflammatory monocytes, macrophages, dendritic cells) between control and Dnm2 wt/K562E mice at 12 weeks of age, both in blood samples of naïve mice (A) and of mice infected with *Listeria monocytogenes* (B), reveals no major changes between genotypes for each cell type. Analysis of the same cell types in spleen (C), bone marrow (D) and peritoneal cavity (E) of infected control and infected Dnm2 wt/K562E animals consistently reveals no major alterations between both genotypes for each cell type. Bar heights, mean; error bars, SEM (*n* = 6 mice/genotype). Two-tailed unpaired Student’s *t*-test with Holm–Sidak’s multiple comparisons test. Significance was set at ^***^P < 0.001.

Some patients harbouring the DNM2 K562E neuropathy-causing mutation also display a mild neutropenia (11). To test whether Dnm2 wt/K562E mice display also signs of neutropenia, we evaluated blood samples of naïve Dnm2 wt/K562E mice and controls for CD4^+^ T cells, CD8^+^ T cells, B cells, neutrophils, inflammatory monocytes, macrophages and dendritic cells (Fig. 8A). We did not detect appreciable changes. Furthermore, we challenged the immune system of Dnm2 wt/K562E and control mice with infection by *Listeria monocytogenes* and analysed immune cells in blood, spleen, bone marrow and peritoneal cavity compartments (Fig. 8B–E). Again, no major alterations indicative of a neutropenia were found, despite a statistically significant but unlikely biologically relevant very small change in B-cell numbers of infected Dnm2 wt/K562E spleens.

## Discussion

In this study, we have analysed mice carrying the equivalent Dnm2 K562E mutation that causes dominantly inherited CMTDIB neuropathy in humans. Somewhat surprisingly, we found no distinct signs of a neuropathy in these animals. In contrast, Dnm2 wt/K562E mice were affected by a mild but definitive and lasting myopathy. These conclusions are supported by behavioural and electrophysiological analyses, together with findings of pathological features affecting skeletal muscles in mutant animals. These attributes include a reduction of muscle fibre diameters, accumulation of macrophages, increased amounts of fibrotic ECM present in muscles and a severely deregulated transcriptome profile of mutant muscle, particularly characterized by decreased expression of transcripts related to mitochondrial oxidative phosphorylation and energy metabolism. Our results indicate that these myopathic features in Dnm2 wt/K562E mice are caused by the Dnm2 K562E allele, since we found no evident signs of a myopathy in Dnm2 wt/0 mice, consistent with a previous complementary analysis (41).

Why do Dnm2 wt/K562E mice fail to show overt signs of a neuropathy, despite genetically mirroring the DNM2 WT/K562E mutation found in CMTDIB? The most obvious explanation relates to differences between mice and humans. Particularly relevant in the context of neuropathies are the much smaller body size of mice and, accordingly, the exceptional lengths of peripheral nerves which make axons vulnerable to distal degeneration (8,42). In agreement, some other mouse models for human neuropathies also do not (or only partially) recapitulate the neuropathology observed in patients (43,44).

The lack of a neuropathy in our Dnm2 wt/K562E mouse model, however, appears to have enabled the fortuitous detection of an otherwise potentially hidden primary myopathy, since an overt neuropathy would have been most likely associated with an overshadowing secondary muscular atrophy. Although we cannot exclude mouse-specific effects of the Dnm2 K562E allele on skeletal muscles, there are some hints that a primary myopathy might contribute to CMTDIB disease also in humans. In particular, a set of DNM2 mutations apparently distinct from those causing CMT are linked to dominantly inherited CNM (22), a muscle disease that is characterized by muscle weakness and myofibre atrophy. However, upon closer inspection, the boundaries between DNM2 mutation-based CNM myopathy and CMT neuropathy become slightly blurred since patients with DNM2 mutation-based CMT neuropathies show also muscle weakness and atrophy due to impaired neural input. In such cases, a mild primary myopathy could potentially remain concealed beneath the expected secondary muscle defects. Of special interest in this context is the description of the DNM2 G359D neuropathy-causing mutation which shows also chronic myopathy-like changes without neurogenic features (45). On the other hand, typical CNM features co-exist with signs of a mild peripheral neuropathy in some patients carrying CNM mutations (46,47). In our current study, we did not find a substantial enrichment of central nuclei in wt/Dnm2 K562E mouse muscles, arguing against a particular relationship with the classical histopathology of CNM mutations. We note, however, that the pathology of central nuclei tends to be more common in humans than in CNM mouse models (48–50). *In vitro*, mutant DNM2 K562E shows reduced GTPase activity and lipid interaction when compared to wild-type DNM2, while at least some CNM mutants tend to be hyperactive (27). Such findings suggest a potential fundamental molecular difference between CMT and CNM mutants that needs to be explored further in appropriate physiological settings with regard to cellular disease mechanisms, taking into account that other factors such as the dynamics of oligomer formation and stability may also contribute (51).

To approach the molecular impact of the Dnm2 K562E allele at the transcriptional level in a global manner, we analysed the transcriptome of soleus muscles of Dnm2 wt/K562E mice compared to controls. The results were in line with the increased ECM deposition and macrophage numbers observed by histological analysis. In addition, we found prominent sets of deregulated genes indicative of mitochondria dysfunction. These findings might reflect either a primary impact of the DNM2 K562E mutation on mitochondrial biology or a more secondary signature associated with the myopathic features observed or both. Interestingly, DNM2 has been directly implicated in mitochondrial dynamics, in particular mitochondrial fission (18). Although this specific issue is controversial (19,20), a potential impact of DNM2 K562E on general mitochondrial biology remains to be examined. On the other hand, gene microarray analyses of models of different types of myopathies have shown downregulation of transcripts related to mitochondrial function as a common feature (52,53). However, detailed and accurate comparisons between our data and the published datasets are limited by the different technologies used and await more detailed studies with comparable state-of-the-art technologies and corresponding bioinformatic analyses.

The histological analysis of a sural nerve biopsy of a DNM2 wt/K562E patient showed myelin abnormalities and focal hypermyelination (11). Such features are usually associated with SC dysfunction and demyelinating neuropathies (54–56). Consistent with these observations, cell culture experiments had suggested that overexpression of mutant Dnm2 K562E can affect myelination by SCs (29). Thus, we analysed the impact of the Dnm2 K562E allele in transgenic mice with an emphasis on its role in SCs. To this end, we studied animals carrying a Dnm2 K562E allele, in which the remaining wild-type allele was inactivated specifically in SCs using the cre/lox technology (P0Cre Dnm2 fl/K562E mice). We found no defects in early peripheral nerve development in such mice, together with normal CME levels of mutant SCs. These findings stand in contrast with the major developmental impairments observed if SCs lack Dnm2 completely (24). Thus, the two mouse models do not show a similar degree of DNM2 loss of function. However, we found also that the P0Cre Dnm2 fl/K562E animals develop a striking neuropathy-like phenotype between 3 and 4 weeks of age. From the later finding, we can conclude that the Dnm2 K562E allele without the presence of a functional Dnm2 allele in SCs cannot sustain peripheral nerves. Whether this effect is due to a loss-of-function or gain-of-function mechanism remains open, although taken all available evidence together, we favour the interpretation that the DNM2 K562E allele is not a full loss-of-function allele, at least in SCs. The interpretation of the lacking phenotype of P0Cre Dnm2 fl/K562E mice in early nerve development remains complex due to technical considerations, combined with the particular biology of dynamin proteins. For example, we cannot rule out that despite the disruption of the wild-type allele in SCs, some wild-type Dnm2 protein that was expressed prior to the genomic disruption may have survived in rather stable dimers or oligomers together with Dnm2 K562E mutant protein. Quantitative examination of such oligomer dynamics, together with establishing the corresponding effects on molecular and cellular functions of DNM2, also in conjunction with potential distinct compensatory roles of other dynamin family members in SCs and muscles (14), remains an experimental challenge for the future.

In summary, we have shown that mice with the corresponding mutation to the CMTDIB neuropathy-causing DNM2 K562E allele show hallmarks of a primary myopathy. The lack of the expected neuropathy in our mouse model may have allowed to unmask this feature that might also be present in neuropathy-affected humans. Testing this hypothesis in patients is probably not straightforward due to the overriding neuropathy and the associated secondary muscle atrophy which is likely to make the determination of a contribution by a primary myopathy quite demanding. Applying techniques involving induced pluripotent stem cells, followed by differentiation to muscle tissue, in combination with CRISPR/Cas-based methods, has the potential to overcome these difficulties in the future (57–59).

## Materials and Methods

### Experimental animals

Mice carrying the *Dnm2^K562E^* point mutation were generated with the Institut Clinique de la Souris (ICS), project number IR3384. In brief, a targeting construct harbouring exon 16 with the nucleotide substitution mimicking the Dnm2 K562E mutation with respective homology arms (4.5 kb for 5′ homology and 3.5 kb for 3′ homology) was used for homologous recombination of mouse ES cells, followed by injection of selected cells into blastocysts. The protamine cre- and selection marker-coding cassettes present in intron 16–17 of the targeting vector were flanked by LoxP sites, which were removed by Cre. From this recombination, one LoxP site, flanked by 44 bp in total, remained present in intron 16–17 and was used for genomic genotype determination by PCR. The resulting chimeric mice were bred further to select for germline transmission of the point mutation. DNA sequencing was used to confirm that the correct mutation was successfully incorporated in the genome of mutant mice, using a sequencing primer annealing in intron 15–16 with the sequence 5′-GAGGCAGAGGCAGACGAATTTC-3′. The DNA submitted for Sanger sequencing was a PCR amplicon generated from genomic DNA of DNM2 wt/K562E mice, including both wild-type and K562E alleles, which was extracted from an agarose gel and purified with a gel clean-up kit (NucleoSpin, Macherey-Nagel). The following primers were used to generate the sequenced amplicon from genomic DNA: forward 5′-GAGGCAGAGGCAGACGAATTTC-3′, reverse 5′-CAGACTTGCCTAGACCGGTCA-3′.

Dnm2 K562E mice were used for most experiments in the heterozygous *Dnm2^+/K562E^* state (designated throughout the manuscript as Dnm2 wt/K562E), unless otherwise indicated in figures or figure legends. Mice were consistently mated with C57bl6/J wild-type animals for colony maintenance. As controls in most experiments, we employed age-matched or littermate *Dnm2^+/+^* mice, with the exception of Figure 3 and Supplementary Material, Figure S3A–J, in which *Dnm2^fl/+^* mice were also used. For transferrin uptake experiments in cells (Supplementary Material, Fig. S3K), the control mice used harboured the *MPZ^Cre^* (Tg(Mpz-cre)26Mes/J; RRID:IMSR_JAX:017927) (designated throughout the manuscript as P0Cre) (37) and the reporter gene *Rosa26-loxPstoploxP-YFP* mouse line (GtROSA26Sor<tm1(EYFP)Cos>; RRID:IMSR_JAX:006148) (identified in the figures by an asterisk next to the genotype) (60), so that SCs could be specifically analysed by FACS.

To achieve conditional excision of one *Dnm2* allele in SCs, we additionally employed *Dnm2^fl/+^* and *MPZ^Cre^* mice (24). These mice were bred together to generate *MPZ^Cre^*:*Dnm2^fl/+^* (designated throughout the manuscript as P0Cre Dnm2 fl/wt) and further bred with the K562E mice to generate *MPZ^Cre^*:*Dnm2^fl/K562E^* (designated throughout the manuscript as P0Cre Dnm2 fl/K562E), in which all mouse cells express the *Dnm2* K562E allele, and SCs of the same mice lack the *Dnm2* wild-type allele.

Mice of either sex were used in the experiments, unless stated otherwise. All mice used are on the C57bl/6 J background. Mice were allocated to experiments based on their age and genotype but otherwise in a random manner. Mice were housed in a maximum of five animals/cage, kept in a 12 h dark–light cycle and provided access to standard chow and water *ad libitum*. Genotypes were determined by genomic PCR, using the following primer sets: DNM2 K562E forward 5′-ACACAGAGCAGAGGTGAGGAAG-3′, reverse 5′-CTAGACCGGTCAGCAGGTTTAG-3′; Cre forward 5′-ATCGCCAGGCGTTTTCTGAGCATAC-3′, reverse 5′-GCCAGATTACGTATATCCTGGCAGC-3′; Dnm2 (floxed and null) forward 5′-GGGAATCCTGCTGGGGAAGCTCTC-3′, Dnm2 floxed reverse 5′-CTCTAGCACTTCCACTAAGCCCTCC-3′, Dnm2 null reverse 5′-GCAGCATGAGACTATGGATCAAGC; Rosa26-loxPstoploxP-YFP forward 5′-AAAGTCGCTCTGAGTTGTTAT-3′, transgenic reverse 5′-GCGAAGAGTTTGTCCTCAACC-3′, and wild-type reverse 5′-GGAGCGGGAGAAATGGATATG-3′. All animal experiments were performed with the approval and in accordance to the guidelines of the Zurich Cantonal Veterinary Office under permits ZH25/2014, ZH129/2011, ZH161/2014 and ZH090/2017.

### Electrophysiology

Electrophysiology analysis was performed as previously described (24,61,62). In brief, Dnm2 wt/K562E and respective control animals were aged to approximately 1 year (11–14 months of age) and weighed prior to the anaesthesia induction. P0Cre Dnm2 fl/K562E mice were aged to between 3 and 4 weeks and weighed prior to the anaesthesia induction. The mice were anaesthetized with a cocktail of ketamine (70–90 mg) and xylazine (7–9 mg) per kg of body weight (adjusted until loss of pedal withdrawal reflex) and placed under a warm lamp to maintain the limb temperature between 32 and 34°C during measurements. Temperature was periodically controlled with an infrared thermometer (Riester). During data acquisition, the examiner was blinded to the genotype of the animals. Supramaximal stimulation (defined as at least 30% above the current required to obtain a maximal amplitude and area under the curve response) was used for both the compound sensory and compound motor analysis. For compound motor analysis, the ‘proximal’ stimulating electrodes were placed at the SN notch, the ‘distal’ stimulating electrodes were placed adjacent to the tibial nerve at the ankle and the detection of compound motor action potentials (CMAP) was achieved with a pair of electrodes in the foot muscles. The motor NCV (mNCV) (in m/s) was calculated with the distal and proximal latencies, considering the distance between these 2 regions. The amplitude of the response reflects the reaction intensity of the foot muscles. The CMAP analysis reflects the response of the motor nerve circuit which stimulates the foot muscles plus the muscle response itself. Repetitive stimulation analysis is a form of CMAP analysis with ‘distal’ stimulation performed as a train of stimuli at 3 Hz and at 10 Hz. Each train consists of 10 successive stimuli, and the analysis of CMAP amplitude changes was based on the comparison of the first and the sixth stimulus.

For compound sensory action potential analysis, the stimulation was performed with the electrodes adjacent to the tibial nerve at the ankle, and the detection was done with the electrodes located at the SN notch. The compound sensory action potential conduction velocity and amplitude reflect the measurement of orthodromic condition by sensory axons and also of antidromic nerve condition by motor axons but is not influenced or dependent on muscle responses.

### Behaviour and gait assessment

Assessment of behaviour included coordination and motor performance tests (rotarod, forepaw grip strength test and, at 2 months of age, also the inverted hang test), a sensory performance test (hot plate) and the open-field arena for overall distance travelled in a 30-min period. Gait was assessed with the CatWalk (Noldus). Rotarod test: Mice were placed on a rod in a Rotarod device (Ugo Basile), which is then accelerated from 4 to 40 rpm. Time spent on the rotating rod was measured. A run is stopped after maximally 10 min, until the mouse dropped from the rod or until the mouse spun twice clinging to the rod. The time that each animal spent on the rod is reported from the average of five trials performed on 1 day per animal, with an intertrial resting period of at least 30 min. Grip strength test: Each mouse was picked up by the base of the tail and lowered towards a horizontal grip bar (diameter ~3 mm) attached to a grip strength metre (Ugo Basile). After grasping the bar with the forelimbs, it was brought to a horizontal position and then gently pulled back until it let go off the bar. The force measured when the animal released the grip was recorded. The animals were tested in five consecutive trials, and the average of peak strength is reported for each animal. Inverted hang test: Mice were placed on top of a grid or standard metal cage-top grid. The grid was gently rocked to stimulate the animal to grip the wires with all four paws. The grid was inverted and hovered above a cage filled with soft bedding. The time until the animal dropped from the grid was recorded, or the animals were removed after 10 min if they did not let go before. Testing sessions for each animal consisted of four trials in 1 day, with an intertrial resting period of at least 30 min. The data is shown as average time across the trials for each mouse. Hot plate: Each mouse was placed on a high-precision plate (Ugo Basile) warmed up to 55°C. The mouse was carefully observed, and the time is measured until the animal licked its hind paws. Open-field exploration test: Just before the test, mice included in a testing session were moved to the testing room and placed in the centre of square plastic chamber arenas. Various chambers (usually four) were imaged simultaneously. Each mouse was allowed to freely explore the chamber environment over a 30 min period of time. The mouse behaviour was recorded as a video. Upon completion of the 30 min testing session, each mouse was returned to its home cage. The videos covering the whole duration of the test were traced with software (Ethovision XT11). Gait assessment with CatWalk: The animals were placed on top of an illuminated glass plate covered by a tunnel and voluntarily moved through the tunnel up to a target box. The contact between the paws and the glass deflects light, which is captured by a camera and registered into a video format. The light deflection allows the software (CatWalk XT 10.6) to calculate parameters regarding gait and locomotion. The data shown is averaged from three compliant runs performed by each mouse. For compliance, the run duration should not be quicker than 0.5 or slower than 5 s, and the maximum allowed speed variation was set at 60%.

### Electron microscopy

Peripheral nerves were extracted and immediately fixed with 3% glutaraldehyde and 4% paraformaldehyde in 0.1 M phosphate buffer. The tissues were kept immersed in the fixative solution at least overnight at 4°C before proceeding. After fixation, the tissue was treated with a 2% osmium tetroxide solution (in 0.1 M phosphate buffer), followed by three washing steps with 0.1 M phosphate buffer, and then dehydrated over an acetone gradient (30, 50, 70, 90, 96% and 2× at 100%). Following dehydration, acetone was replaced by Spurr’s resin (Electron Microscopy Sciences) over sequential treatments with a resin/acetone gradient (33, 50, 66% and 2× 100%). Ultrathin sections were prepared with a UC7 ultramicrotome (Leica) or a Reichert-Jung Ultracut E ultramicrotome (Leica). For imaging of entire cross-section surface at high resolution (concerns all EM images shown and quantified in this manuscript, except those on Supplementary Material, Fig. S1), ultrathin sections were prepared at 99 nm and deposited in indium tin oxide (ITO) coverslips (Optics Balzers) and imaged with the in-lens detector of a Zeiss Merlin scanning electron microscope attached to an ATLAS module. Data acquisition was controlled via the ATLAS software (versions 4, 5). To generate the images shown and quantified in Supplementary Material, Figure S1, ultrathin sections were prepared with 65 nm thickness and deposited in copper grids (Electron Microscopy Sciences, G200-Cu), and random areas were imaged with a FEI Morgagni 268 TEM at high magnification. Myelin periodicity values obtained are in line with previous measurements on aldehyde-fixed samples (63).

### Morphological analysis and g-ratio measurements

The number of myelinated axons, sorted axons, axons without myelin and myelin aberrations were counted manually using Photoshop count tool (Adobe, versions CS6 or CC) on the EM panoramas covering the entire cross-section surface of the nerve for each mouse. To calculate the axonal diameter of all axons (without myelin abnormalities) on the distal tibial nerve, the area of each axon was measured, and the diameter was derived from the area. To calculate g-ratios (axonal diameter/fibre diameter), measurements were performed in at least three randomly selected regions of each EM panorama, and all axons without myelin abnormalities within these regions were quantified. Within these locations, the area of each axon (without myelin abnormalities) was measured and the axonal diameter derived from the area. To calculate the fibre diameter, the axonal diameter was added to the average thickness of myelin measured at two different and histologically well-preserved locations of the respective myelin ring. At least 97 fibres were measured per genotype.

### Antibodies and chemicals

The following primary antibodies were used: laminin (Sigma Aldrich, #L9393, RRID:AB_477163, 1:200), CD68 (Bio-Rad, #MCA1957, RRID:AB_322219, 1:100), synaptophysin (GeneTex, #100865, RRID:AB_2038077, 1:500) and embryonic myosin (DSHB, #F1.652, RRID:AB_528358, 2 μg/ml). Analysis of muscle fibre types was performed with isotype-specific mouse primary antibodies: myosin heavy chain type I (DSHB, #BA-D5, RRID:AB_2235587, 2 μg/ml), myosin heavy chain type IIA (DSHB, #SC-71, RRID:AB_2147165, 2 μg/ml) and myosin heavy chain type IIB (DSHB, #BF-F3, RRID:AB_2266724, 2 μg/ml). To detect each primary immunoglobulin isotype in the fibre-type analysis, the following secondary antibodies were used: mouse IgG2b (Thermo Fisher Scientific, #A-21242, RRID:AB_2535811, 1:500), mouse IgM (Thermo Fisher Scientific, #A-21045, RRID:AB_2535714, 1:500) and mouse IgG1 (Thermo Fisher Scientific, #A-21121, RRID:AB_2535764, 1:500). Analysis of immune cells with FACS employed the following fluorescently labelled primary antibodies: CD8a (Thermo Fisher Scientific, #11–0081-81, RRID:AB_464914, 1:200), TCRβ-PE (BioLegend, #109208, RRID:AB_313431, 1:400), CD11b-PerCP-Cy5.5 (BioLegend, #101228, RRID:AB_893232, 1:800), Gr-1-APC (Thermo Fisher Scientific, #17–5931-82, RRID:AB_469476, 1:4000), CD19-PE-Cy7 (BioLegend, #115520, RRID:AB_313655, 1:2000), CD45-BV785 (BioLegend, #103149, RRID:AB_2564590, 1:1000), CD4-BV711 (BioLegend, #100447, RRID:AB_2564586, 1:600), CD11c-BV605 (BioLegend, #117333, RRID:AB_11204262, 1:500) and Ly-6G-BV421 (BioLegend, #127628, RRID:AB_2562567, 1:800). Fluorophore-coupled secondary antibodies used in the other experiments of this study were purchased from Jackson ImmunoResearch and Life Technologies and used at 1:500 dilution for immunostainings. Alexa488-coupled α-bungarotoxin (B13422) was purchased from Thermo Fisher Scientific.

### Whole-mount immunostainings of neuromuscular junctions

Soleus muscles were extracted and fixed with insect pins on Sylgard-coated plates (Sigma Aldrich, #761036-5EA). Approximately 100 μl of Alexa488-coupled α-bungarotoxin (5 μg/ml in PBS) was gently injected within the muscle, which loosens the packaging of the myofibres and facilitates diffusion, and incubated for 30 min at room temperature (RT). Following incubation and three washes with PBS, the soleus muscles were chemically fixed with 4% PFA diluted in PBS for 15 min at RT. Following three PBS washes, the muscles were gently separated with in small bundles of myofibres, followed by permeabilization with 1% Triton X-100 (Sigma Aldrich, #X100) for 30 min at RT. Residual free PFA was neutralized with 100 mm glycine in PBS for 15 min at RT. After permeabilization, muscle bundles were immersed in blocking buffer (1% BSA and 10% goat serum in PBS) for 30 min and incubated with the synaptophysin antibody (GeneTex, #100865, RRID:AB_2038077, 1:500), diluted in blocking buffer, overnight at 4°C. The following morning, the tissue was washed four times with blocking solution over a period of 2 h at RT. Fluorophore-coupled secondary antibodies were diluted in blocking buffer and incubated with the muscle bundles for 45 min at RT. Tissues were finally washed four times with blocking solution over a period of 2 h at RT and mounted with VECTASHIELD antifade mounting media (Vector lab #H-1000). Stacks of images were acquired over several z focus plans using an epifluorescence Zeiss Axio Imager M2 microscope equipped with a motorized stage and monochromatic CCD camera (sCMOS, pco.edge), via the Zeiss Zen 2 software (blue edition). The stacks of images were converted into flat maximum intensity projections, which were quantified for endplate innervation by assessing whether the postsynaptic α-bungarotoxin signal had complete, partial or no overlap with the presynaptic synaptophysin signal.

### Section preparation and immunostainings of muscle tissue

Soleus or TAs were quickly extracted and embedded in a 7% tragacanth (Sigma #G1128) slur. The tragacanth with the muscle was placed on top of a cork circle and immersed for 20 s in the liquid fraction of semi-frozen isopentane (cooled with liquid nitrogen). After freezing, samples were quickly moved to dry ice and then frozen at −80°C until further use. Frozen sections were prepared with a rotary cryotome (Hyrax 60, or CryoStar NX70) at 10 μm thickness and collected on microscopy slides with superfrost coating (Menzell-Gläser). Microscopy slides with the sections were used for immunostainings and also for the H&E and Masson’s trichrome staining (see below). For immunostainings, microscopy slides were immersed in blocking buffer (10% goat serum in PBS) for 30 min at RT. Primary antibodies were diluted in blocking buffer (see *Antibodies and chemicals* section of the material and methods for antibody serial number and dilution) and incubated with the tissue sections inside a moist-chamber for 1 h at RT, followed by three washes of 5 min with PBS. Secondary antibodies were diluted in blocking buffer and incubated for 45 min at room temperature with the sections, protected from light, followed by three washes for 5 min with PBS before mounting with fluorescence mounting media. Some immunostainings were counterstained with DAPI to label nuclei. Note that the immunostainings of CD68 and laminin on different tissues and time points shown in Figures 4, 6 and 7 were performed several months apart from each other and are best comparable between control and mutants within each experiment (which were handled simultaneously). Images were acquired using an epifluorescence Zeiss Axio Imager M2 microscope equipped with a motorized stage and monochromatic CCD camera (sCMOS, pco.edge), via the Zeiss Zen 2 software (blue edition).

### H&E staining of muscle tissue

Microscopy slides with tissue sections were removed from the freezer and air-dried at RT. Slides were then immersed in haematoxylin solution according to Mayer (Sigma Aldrich, #51275) for 1 min and 30 s, followed by washing under running tap water for 2 min. Sections were then immersed in eosin solution (Sigma Aldrich, #HT110332) for 50 s and washed again under running tap water for 2 min. Sections were treated with an incremental gradient of ethanol (1 min each, 70%, 2× 95%, 2× 100%), followed by 5 min immersion in xylene. Microscopy slides were finally mounted with Entellan (Merck #1079600500). Imaging of H&E and Masson’s trichrome staining (see below) was performed using a Zeiss Axioplan2 imaging microscope, equipped with a colour camera (Zeiss Axiocam color HR), and via the Axiovision software (release 4.8).

### Masson’s trichrome staining of muscle tissue

Microscopy slides with tissue sections were removed from the freezer and air-dried at RT and then immersed for 5 min in distilled water. Sections were immersed in preheated Bouin’s solution (Sigma #HT10132) for 15 min at 56°C and then washed under tap water at RT. Sections were then immersed in haematoxylin solution (Sigma Aldrich, #51275) for 5 min, washed for 5 min under running tap water and rinsed with distilled water. Sections were then stained with Biebrich scarlet-acid fuchsin solution (Sigma Aldrich, #HT15) for 5 min and rinsed with distilled water. Microscopy slides were immersed into phosphotungstic/phosphomolybdic acid working solution (Sigma Aldrich, #HT15) for 5 min and then moved into aniline blue solution (Sigma Aldrich, #HT15) for another 5 min, followed by acetic acid (1%) treatment for 2 min, and were then rinsed with distilled water. After the staining, sections were treated with an incremental gradient of ethanol (1 min each, 70%, 2× 95%, 2× 100%), followed by 5 min immersion in xylene. Microscopy slides were finally mounted with Entellan (Merck #1079600500).

### Preparation of primary mouse SCs and transferrin uptake assay

SNs were dissected from P2 to P4 P0Cre Dnm2 control^*^, P0Cre Dnm2 wt/K562E^*^ and P0Cre Dnm2 fl/K562E^*^ pups (five individual animals per genotype), and the perineurium with overlaying epineurium was removed as much as possible. The tissue was enzymatically treated with 1.25 mg/ml trypsin (Sigma Aldrich, #T9201) and 2 mg/ml collagenase (Sigma Aldrich, #C0130) for 1 h at 37°C. Samples were then centrifuged and resuspended as dissociated cells in DMEM–GlutaMAX with 10% FCS (Life Technologies) and seeded in 3.5 cm PLL-coated plates at a density of 150 000 cells/plate and cultured overnight. Prior to labelled transferrin uptake assay, the cells were serum-starved in DMEM GlutaMAX for 30 min at 37°C. The cells were incubated with 10 μg/ml of transferrin labelled with Alexa Fluor 568 (Thermo Fisher Scientific, #T23365) for 3 min at 37°C to allow endocytic uptake. To define the fluorescence background levels, we treated cells as described above but kept them for 3 min at 4°C instead of 37°C to prevent transferrin uptake. Subsequently, cells were quickly transferred to ice to halt transferrin uptake, washed 2× with pre-cooled PBS, treated with a solution (0.2 M Na2HPO4, 0.1 M citric acid) for 2 min to strip away non-internalized transferrin and washed again twice with pre-cooled PBS. Cells were then trypsinized, centrifuged and resuspended in 400 μl pre-cooled flow buffer (PBS, 2% BSA, 5 mm EGTA) and kept cooled on ice until analysed. Transferrin fluorescence levels in each cell were analysed with a SONY SH800 FACS machine. Data analysis was performed with the FlowJo software (RRID:SCR_008520, version 10.0.7). SCs were first gated according to forward and side scatter, followed by selection for the recombination marker YFP to ensure only SCs were analysed. Transferrin fluorescence was measured in more than 1800 cells per sample in most samples, with an exception of one sample in which 253 cells were analysed. The values of the sample with less cells were similar to values of other samples with the same genotype. To account for differences in signal intensity for each sample, the flow cytometry profiles were normalized.

### RNA extraction for RNA sequencing

Soleus muscles were dissected from four independent male mice per genotype at 2 months of age. The genotypes analysed include controls (Dnm2^+/+^) and Dnm2 K562E point-mutant mice (Dnm2^+/K562E^). The muscles were snap frozen in liquid nitrogen immediately after extraction and mechanically grinded while frozen using an RNAse free pestle (VWR international, #4310094) to produce smaller chunks and frozen tissue powder. RNA extraction was performed using Qiazol (Qiagen) as per manufacturer’s instructions. The samples were used for RNA sequencing (see below).

### Illumina RNA sequencing

#### Library preparation

The quality of isolated RNA was determined with a **Qubit**^**®**^**(1**.**0) Fluorometer (Life Technologies**, California, USA) and a Bioanalyzer 2100 (Agilent). Only those samples with a 260 nm/280 nm ratio between 1.8 and 2.1, and a 28S/18S ratio within 1.5–2, were further processed. The TruSeq RNA Sample Prep Kit v2 (Illumina) was used in the succeeding steps. Briefly, total RNA samples (100–1000 ng) were poly A enriched and then reverse-transcribed into double-stranded cDNA. The cDNA samples were fragmented, end-repaired and polyadenylated before ligation of TruSeq adapters containing the index for multiplexing fragments containing TruSeq adapters on both ends were selectively enriched with PCR. The quality and quantity of the enriched libraries were validated using **Qubit**^**®**^**(1**.**0) Fluorometer and the Caliper GX** LabChip^®^ GX (**Caliper Life Sciences)**. The product is a smear with an average fragment size of approximately 260 bp. **The libraries were normalized to 10 nM in** Tris–Cl 10 mm, pH 8.5, with 0.1% Tween 20.

#### Cluster generation and sequencing

**The TruSeq PE Cluster Kit HS4000 or TruSeq SR Cluster Kit HS4000** (Illumina) were used for cluster generation using 10 pm of pooled normalized libraries on the cBOT. Sequencing was performed on the Illumina HiSeq 4000, as paired end at 2 X151 bp using the **TruSeq SBS Kit HS4000** (Illumina).

#### Treatment of sequencing reads

The raw reads were first cleaned by removing adapter sequences, trimming low quality ends and filtering reads with low quality (phred quality < 20) using Trimmomatic (Version 0.33) (64). The read alignment was done with STAR (v2.5.1b) (65). As a reference, we used the Ensembl *Mus musculus* reference genome (build GRCm38.p5) with the gene annotations downloaded on 2015-06-25 from Ensembl (release 80). The STAR alignment options were ‘--outFilterType BySJout --outFilterMatchNmin 30 --outFilterMismatchNmax 10 --outFilterMismatchNoverLmax 0.05 --alignSJDBoverhangMin 1 --alignSJoverhangMin 8 --alignIntronMax 1000000 --alignMatesGapMax 1000000 --outFilterMultimapNmax 50’. Gene expression values were computed with the function featureCounts from the R package Rsubread (v1.22.3) (66). The options for featureCounts were —min mapping quality 10—min feature overlap 10 bp—count multi-mapping reads—count only primary alignments—count reads also if they overlap multiple genes.

To detect differentially expressed genes, we applied a count-based negative binomial model implemented in the software package EdgeR (R version: 3.3.2, EdgeR version: 3.14.0) (67). The differential expression was assessed using an exact test adapted for overdispersed data. Genes showing altered expression with adjusted (Benjamini and Hochberg method) *P*-value < 0.05 were considered significantly regulated. From this set of genes, we considered a log2 fold change of ±0.58 (approximately equivalent to ±1.5 fold change in a linear scale) to generate lists of differentially upregulated or downregulated transcripts between Dnm2 control and Dnm2 wt/K562E animals. These lists were separately subjected to Gene Ontology analysis of biological processes (the downregulated transcripts were further analysed with Gene Ontology of cellular components) using the online tool Enrichr (2015 version, http://amp.pharm.mssm.edu/Enrichr/). An orthogonal analysis using a single condensed list containing the upregulated and downregulated transcripts was also performed with IPA Ingenuity (2017, Qiagen).

### Evaluation of lymphocytes

#### Listeria monocytogenes infection

*Listeria monocytogenes* was grown in brain–heart infusion broth to mid-log phase, and the number of bacteria was estimated by measuring the optical density at 600 nm. Control and Dnm2 wt/K562E littermate and age-matched mice at 12 weeks of age were infected by intraperitoneal injection with 2 × 10^5^ CFU *Listeria monocytogenes*. The injected bacterial number was determined upon overnight culture at 37°C of a sample from the bacterial suspension on brain–heart infusion agar plates. Mice were euthanized and analysed 16 h after infection.

#### Cell preparation for flow cytometric analysis

Blood samples were incubated with ACK lysis solution to remove erythrocytes. Single-cell suspension of splenic cells was obtained by gently pressing spleens through 70 μm cell strainers (Corning, cat. no. 352350). Bone marrow cells were harvested from one femur per mouse. Peritoneal lavage was performed by repeatedly flushing the peritoneal cavity with 10 ml PBS/2% FCS. Single-cell suspensions from the different tissues were resuspended in PBS/2% FCS and briefly incubated with Fc blocking antibodies (clone 2.4G2, purified in-house). Cells were then stained with the following fluorescently labelled monoclonal antibodies: anti-CD8α-FITC (ThermoFisher, cat. no. 11-0081-81), anti-TCRβ-PE (BioLegend, cat. no. 109208), anti-CD11b-PerCP-Cy5.5 (BioLegend, cat. no. 101228), anti-Gr-1-APC (ThermoFisher, cat. no. 17-5931-82), anti-CD19-PE-Cy7 (BioLegend, cat. no. 115520), anti-CD45-BV785 (BioLegend, cat. no. 103149), anti-CD4-BV711 (BioLegend, cat. no. 100447), anti-CD11c-BV605 (BioLegend, cat. no. 117333) and anti-Ly-6G-BV421 (BioLegend, cat. no. 127628). For nonviable cell exclusion, Fixable Viability Dye eFluor^®^ 780 (ThermoFisher, cat. no. 65-0865-18) was used. Stained cells were then fixed with 4% formalin and resuspended in PBS/2% FCS for flow cytometric analysis using a LSRFortessa (BD Biosciences). FACS data were then analysed with FlowJo software (RRID:SCR_008520, version 10.0.7).

### Histological quantifications and preparation of figure panels

Quantification of features on optical and EM images was performed using Adobe Photoshop (version CS6 and CC). Figure levels were adjusted in the same manner in controls and mutants to better visualize the features. Figure panels were assembled with Adobe Illustrator (version CC). Quantifications were statistically processed as indicated below.

### Statistical analysis

Data processing and statistical analyses were performed using GraphPad Prism (RRID:SCR_002798, versions 7 and 8) and Microsoft Excel (version 16.23). Data distribution was assumed to be normal, and variances were assumed to be equal, although this was not formally tested due to low *n* number. Sample sizes were chosen according to sample sizes generally employed in the field. The investigators were formally blinded to the genotypes during analysis of morphological and immunohistochemical data and during recording of behaviour and electrophysiology data. No randomization methods were used. Two-tailed unpaired Student’s *t*-test was used if only two conditions or genotypes were compared. When one of these two conditions was a constant, then one-sample *t*-test was used instead. When more than two conditions were directly compared, one- or two-way ANOVAs followed by Tukey’s or Sidak’s multiple comparisons tests were employed, as indicated in the figure legends and statistics summary file (Supplementary Material, Fig. S8). *P* < 0.05 was considered to be statistically significant. No samples or data were omitted during the analyses.

## Author Contributions

Conceptualization: J.A.P., J.G., M.G., D.G., E.T., S.L., M.A.R., M.K., K.V.T. and U.S. Investigation: J.A.P., J.G., M.G., D.G., L.T., A.O., S.B., F.S., E.T. and K.V.T. Formal analysis: J.A.P., J.G., M.G., D.G., L.T., K.V.T. and U.S. Data Curation: J.A.P., J.G., L.T., A.O., S.B., K.V.T. and U.S. Methodology: J.A.P., J.G., M.G., D.G., L.T., F.S., S.L., M.A.R., M.K. and K.V.T. Visualization: J.A.P. and J.G. Validation: J.A.P., J.G., L.T., A.O. and K.V.T. Resources: M.K. and U.S. Supervision: U.S. Funding acquisition: U.S. Project administration: U.S. Writing original draft: J.A.P. Review and editing the manuscript: J.A.P., J.G., M.G., D.G., L.T., A.O., S.B., F.S., E.T., S.L., M.A.R., M.K., K.V.T. and U.S.

## Data Availability

RNA sequencing data have been deposited in the GEO database under accession number GSE134488.

## Supplementary Material

Pereira_HMG-2020-D-0040_Revised_Supplemental_Data_ddaa034
