# Supplementary material for: Mice carrying an analogous heterozygous dynamin 2 K562E mutation that causes neuropathy in humans develop predominant characteristics of a primary myopathy
Source: Hum Mol Genet. 2020 Mar 4;29(8):1253–73. doi: 10.1093/hmg/ddaa034 (PMC7254847; doi:10.1093/hmg/ddaa034)
Supplement: Pereira_HMG-2020-D-0040_Revised_Supplemental_Data_ddaa034 [file pereira_hmg-2020-d-0040_revised_supplemental_data_ddaa034.pdf]

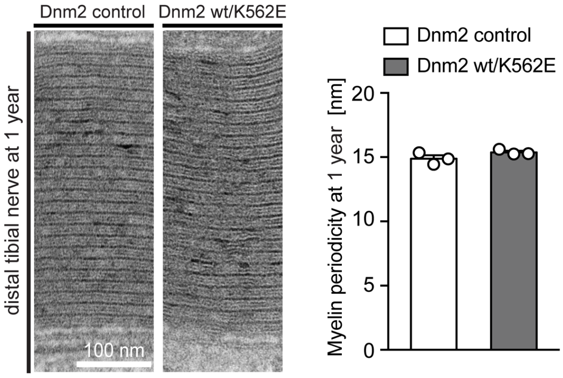

Supplementary Material Figure S1: Normal myelin periodicity in Dnm2 wt/K562E mice. Exemplary EM pictures of myelin in distal tibial nerves from samples of 1 year-old control and Dnm2 wt/K562E mice were analysed for myelin periodicity, which is not significantly altered between both genotypes. Scale bar: 100nm, refers to whole panel. Bar heights: Mean; error bars: s.e.m. (n = 3 mice/genotype, 10 myelin sheaths per animal were analysed and averaged). Two-tailed unpaired Student's t-test.

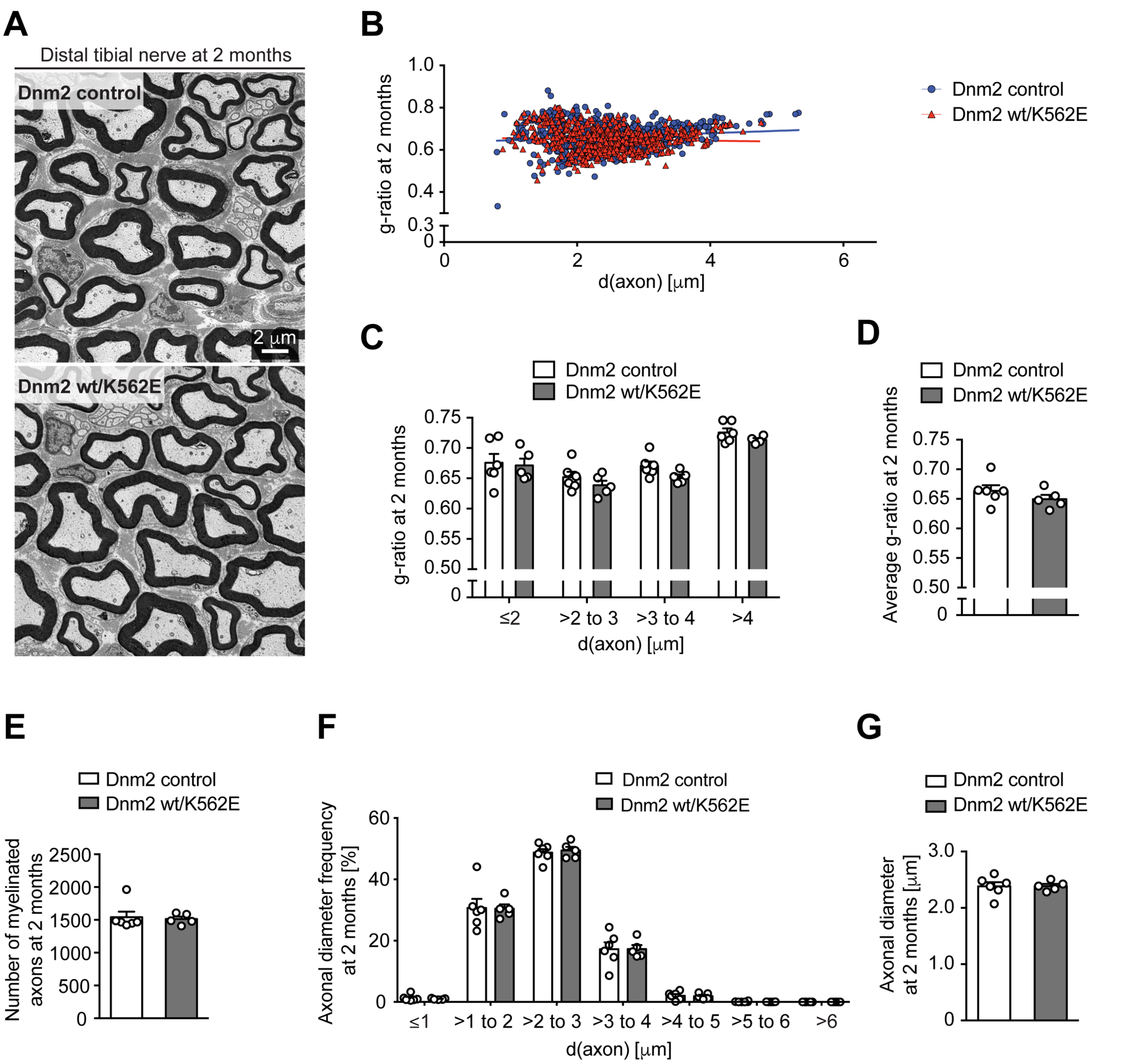

Supplementary Material Figure S2: No overt histological signs of a neuropathy on distal tibial nerves of Dnm2 wt/K562E mice at 2 months of age.

(**A**) Exemplary EM pictures of distal tibial nerve cross sections of control and Dnm2 wt/K562E animals at 2 months. Scale bar: 2µm, refers to whole panel. (**B**) Scatter plot of g-ratio versus axonal diameter from measurements performed on distal tibial nerve EM micrographs derived from 2 months-old mice. Each datapoint represents one axon (n = 6 control and n = 5 Dnm2 wt/K562E mice, at least 97 axons were quantified per animal). (**C, D**) Binned g-ratio distribution per axonal diameter (**C**), and average g-ratio (**D**), of distal tibial nerves derived from 2 months-old mice (same nerves analysed as depicted in **B**). No significant differences were detected between g-ratios of both genotypes. Bar heights: Mean; error bars: s.e.m. (n = 6 control and n = 5 Dnm2 wt/K562E mice). (**E to G**) Number of myelinated axons (**E**), frequency distribution of axonal diameter bins (**F**), and average axonal diameter (**G**). All myelinated axons (**E**), and myelinated axons without myelin abnormalities (**F, G**), were measured in EM micrographs covering the entire cross-section of distal tibial nerves derived from 2 months-old mice. No

systematic changes in axonal diameters between Dnm2 wt/K562E and controls were detected. Bar heights: Mean; error bars: s.e.m. (n = 6 control and n = 5 Dnm2 wt/K562E mice). Two-Way ANOVA with Sidak´s multiple comparisons test (**C, F**), two-tailed unpaired Student's t-test (**D, E, G**).

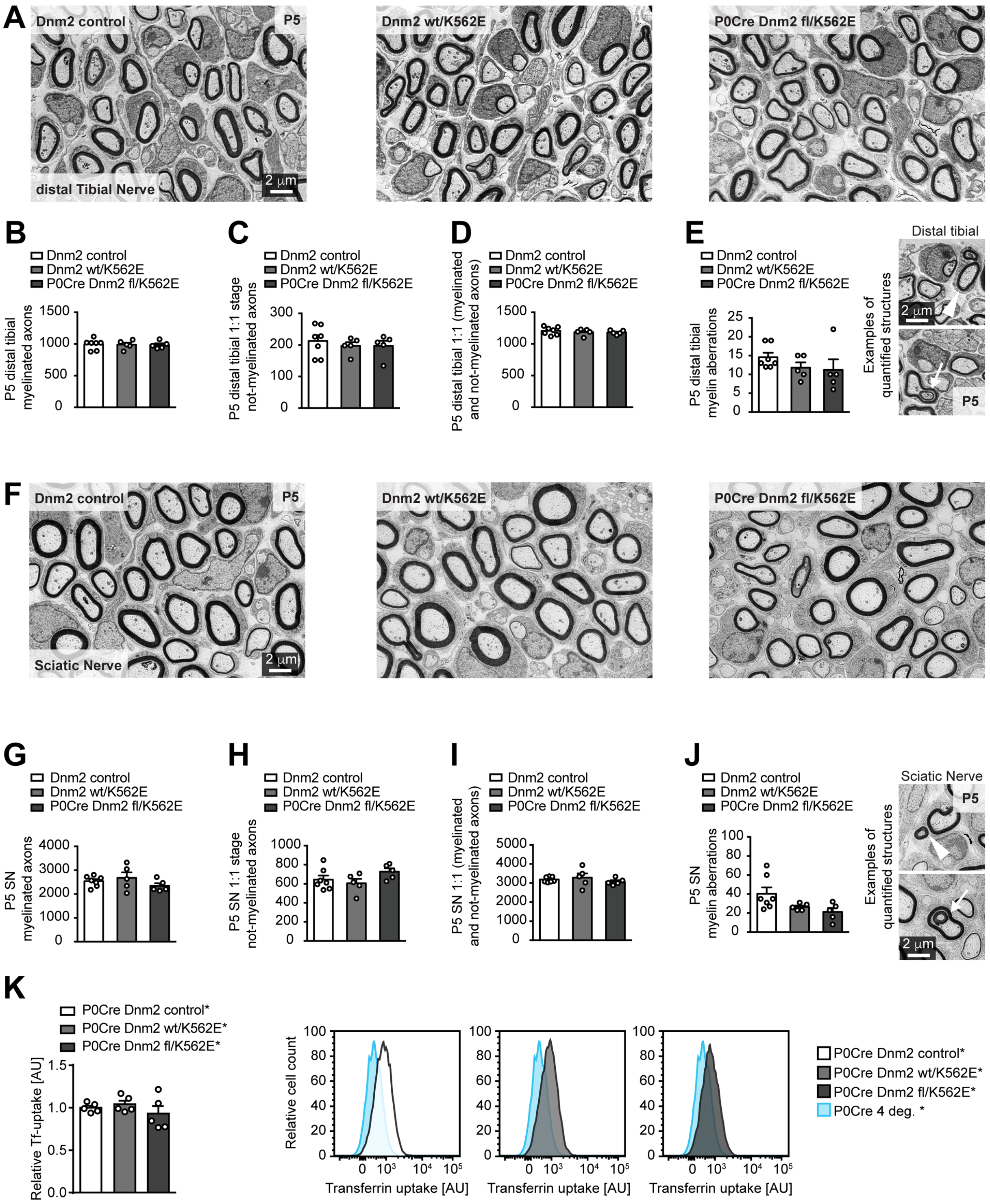

Supplementary Material Figure S3: Excision of the wildtype allele from Schwann cells of DNM2 K562E-expressing mice does not significantly impair axon sorting and onset of myelination at P5.

(**A**) Exemplary EM pictures of cross sections of distal tibial nerves of control, Dnm2 wt/K562E, and P0Cre Dnm2 fl/K562E at P5. Scale bar: 2µm, refers to whole panel. (**B to D**) Quantification of myelinated axons (**B**), and not-myelinated 1:1 SC-axon profiles (**C**) reveals no differences on the onset of myelination between control, Dnm2 wt/K562E and P0Cre Dnm2 fl/K562E distal

tibial nerves at P5. The total number of sorted axons (**D**, includes myelinated and not-myelinated 1:1 profiles) indicates no alterations on the outcome of radial sorting between control, Dnm2 wt/K562E and P0Cre Dnm2 fl/K562E distal tibial nerves. Whole cross-section EM panoramas were used for quantifications. Bar heights: Mean; error bars: s.e.m. (n = 7 control, n = 5 Dnm2 wt/K562E and P0Cre Dnm2 fl/K562E animals). (**E**) Evaluation of myelin abnormalities including outfoldings (white arrowhead) and infoldings (white arrow) throughout the entire cross-section of P5 distal tibial nerves in EM panoramas reveals no significant differences different between control, Dnm2 wt/K562E, and P0Cre Dnm2 fl/K562E mice. The images depicting examples of quantified structures derive from Dnm2 wt/K562E mice. Scale bar: 2μm, refers to whole panel. Bar heights: Mean; error bars: s.e.m. (n = 7 control, n = 5 Dnm2 wt/K562E and P0Cre Dnm2 fl/K562E animals). (**F**) EM micrographs of sciatic nerve cross sections of control, Dnm2 wt/K562E, and P0Cre Dnm2 fl/K562E at P5. Scale bar: 2μm, refers to whole panel. (**G to I**) Quantification of myelinated axons (**G**), and 1:1 not-myelinated SC-axon profiles (**H**) shows no differences in the onset of myelination between control, Dnm2 wt/K562E and P0Cre Dnm2 fl/K562E sciatic nerves at P5. The total number of sorted axons (**I**, includes myelinated and 1:1 not-myelinated) indicates no alterations on the outcome of radial sorting between control, Dnm2 wt/K562E and P0Cre Dnm2 fl/K562E sciatic nerves. Whole cross-section EM panoramas were used for quantifications. Bar heights: Mean; error bars: s.e.m. (n = 7 control, n = 5 Dnm2 wt/K562E and P0Cre Dnm2 fl/K562E animals). (**J**) Evaluation of myelin abnormalities including outfoldings (white arrowhead) and infoldings (white arrow) throughout the entire cross-section of P5 sciatic nerves in electron micrograph panoramas reveals no significant differences between control, Dnm2 wt/K562E and P0Cre Dnm2 fl/K562E mice. The images depicting examples of quantified structures derive from Dnm2 wt/K562E mice. Scale bar: 2μm, refers to whole panel. Bar heights: Mean; error bars: s.e.m. (n = 7 control, n = 5 Dnm2 wt/K562E and P0Cre Dnm2 fl/K562E animals). (**K**) FACS analyses of transferrin (Tf) uptake of YFP+ primary mouse SCs derived from control*, P0Cre Dnm2 wt/K562E*, and P0Cre Dnm2 fl/K562E*. Control SCs incubated at 4°C defines the baseline of the experiments. A profile of the 4°C condition is shown next to exemplary profiles derived from individual genotypes (right side). All genotypes contained P0Cre and the reporter ROSA eYFP (indicated by an *****) to ensure the FACS analysis was exclusive to SCs. (independent Schwann cell preparations were performed from every mouse, n = 5 mice/genotype). One-Way ANOVA with Tukey´s multiple comparisons test.

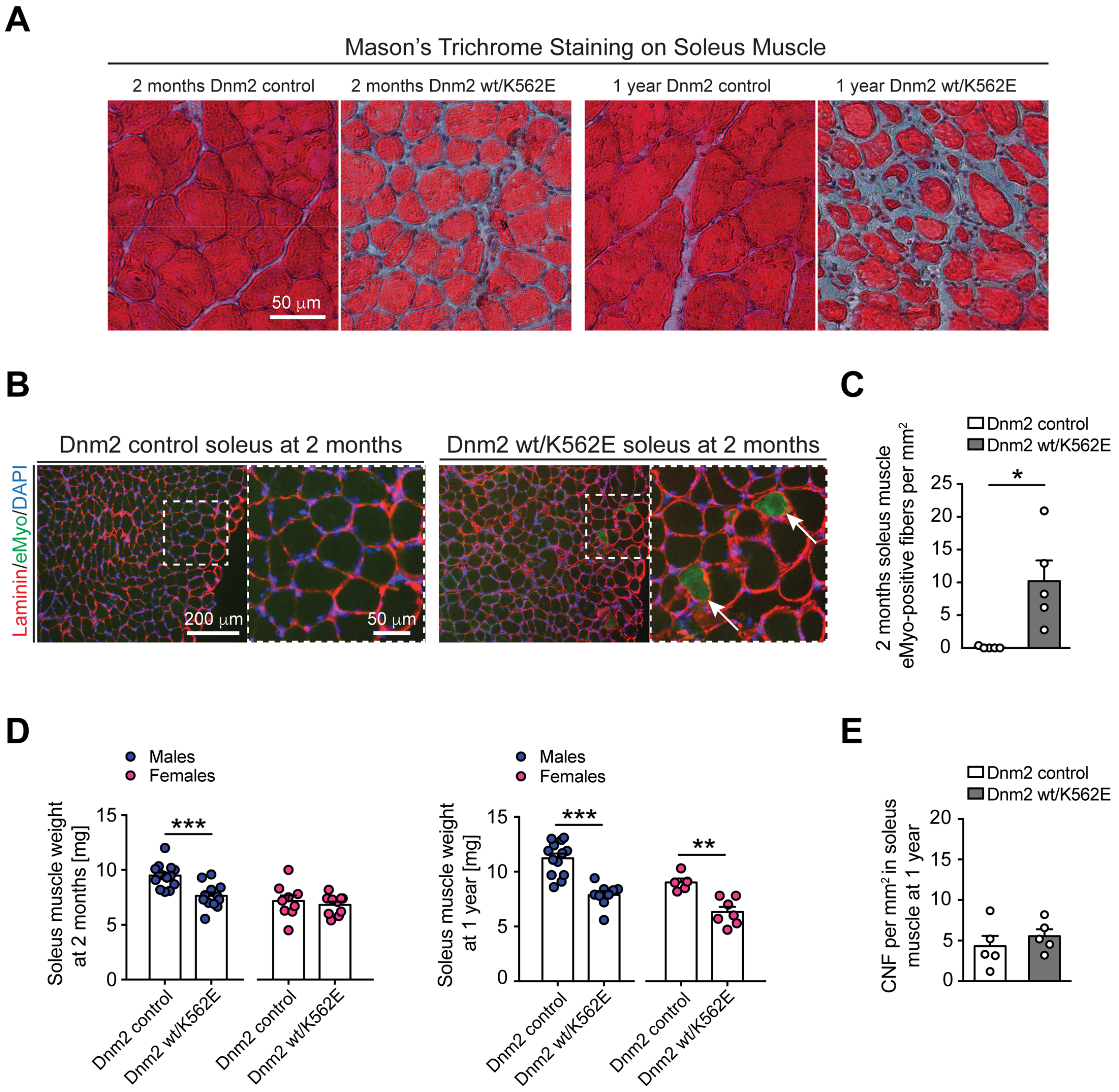

Supplementary Material Figure S4: Additional evidence for a myopathy in Dnm2 wt/K562E soleus muscles.

(**A**) Exemplary pictures of Mason's Trichrome-stained soleus muscle cryosections derived from control and Dnm2 wt/K562E mice at 2 months- and 1 year of age. Dnm2 wt/K562E soleus muscles display a wider extracellular-stained area in the mutant compared to control samples. (n = 5 mice per genotype, at least 3 pictures per mouse were acquired). Scale bar: 50µm, refers to whole panel. (**B, C**) Exemplary cryosections of soleus muscles from control and Dnm2 wt/K562E mice at 2 months of age probed with antibodies in (**B**) targeting laminin or embryonic myosin isoform (eMyo), and stained with DAPI. Exemplary eMyo-positive myofibers (white arrows) are highlighted. Scale bar: 200µm for the lower magnification images in the panel, and 50µm for the magnified images in the panel. Quantification of eMyo-positive myofibers (**C**) shows a significant increase in Dnm2 wt/K562E soleus muscles compared to controls. Bar

heights: Mean; error bars: s.e.m. (n = 5 mice/genotype, images from at least 3 sections quantified and averaged per mouse). (**D**) Analysis of soleus muscle weight (including the calcaneal tendon, used for handling the tissue) of control and Dnm2 wt/K562E sex-specific mice at 2 months- and 1 year of age. The muscles have a lower weight in Dnm2 wt/K562E males and females if compared to the respective controls at 1 year, a feature that is also significant in males at 2 months. Bar heights: Mean; error bars: s.e.m. (females at 2 months: n = 9 control and n = 10 Dnm2 wt/K562E; females at 1 year: n = 5 control and n = 7 Dnm2 wt/K562E; males at 2 months: n = 14 control and n = 12 Dnm2 wt/K562E; males at 1 year : n = 13 control and n = 9 Dnm2 wt/K562E). (**E**) Analysis of centronucleated fibers (CNF) in control and Dnm2 wt/K562E soleus muscle cryosections derived from 1 year-old mice reveals no significant changes between both genotypes. Bar heights: Mean; error bars: s.e.m. (n = 5 mice/genotype, images from 3 sections quantified and averaged per mouse). Two-tailed unpaired Student's t-test. Significance was set at *$p < 0.05$, **$p < 0.01$, ***$p < 0.001$.

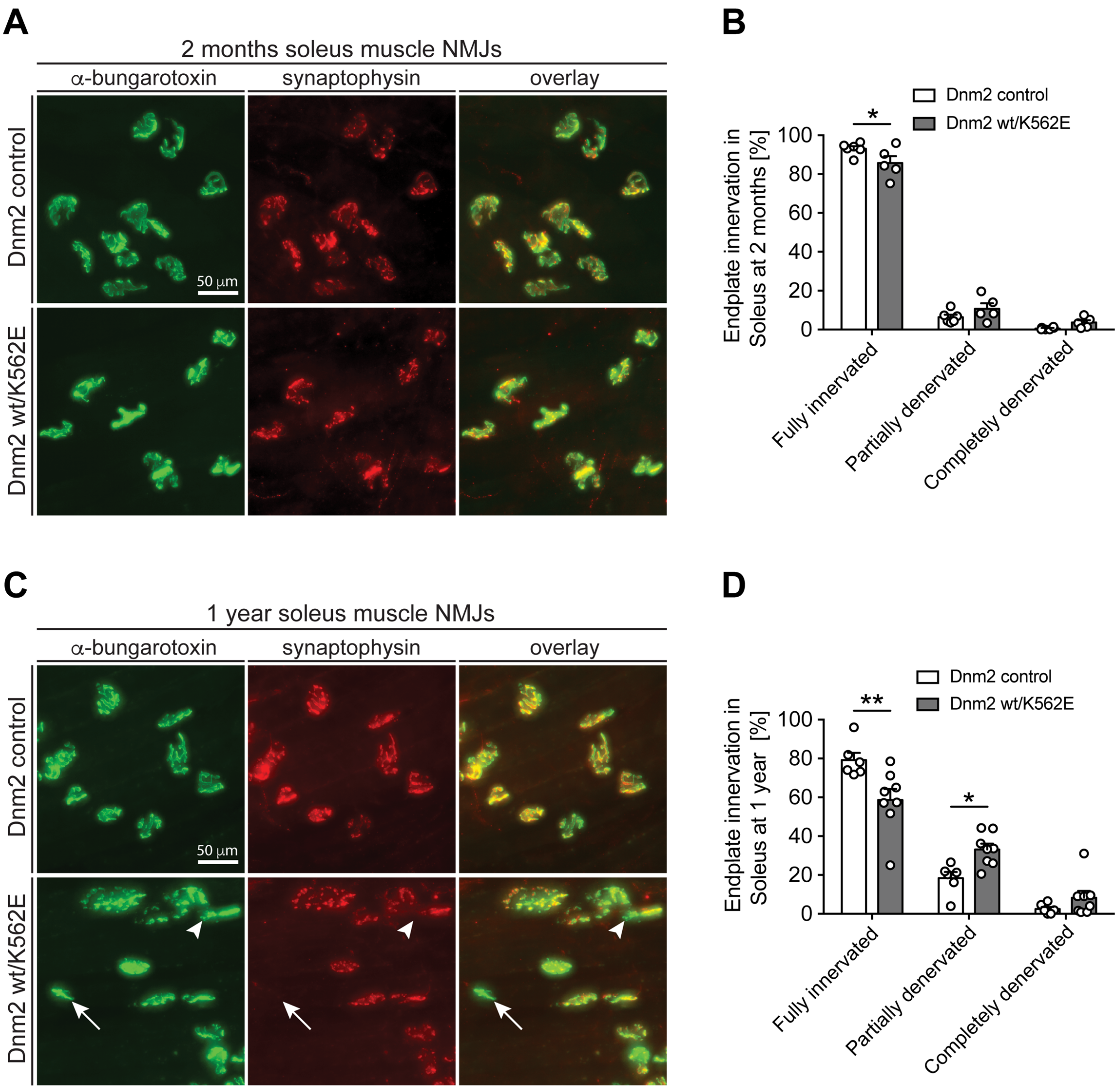

Supplementary Material Figure S5: Neuromuscular junction analysis of Dnm2 wt/K562E soleus muscle does not show a significant increase in completely denervated endplates.

(**A, C**) Exemplary images from whole-mount soleus muscle fibers with labelled neuromuscular junctions (NMJs) post-synaptic (α-bungarotoxin) and pre-synaptic (synaptophysin) terminals derived from mice at 2 months (**A**) and 1 year of age (**C**). Examples of post-synaptic signals that are partially (white arrowhead) or completely (white arrow) not overlaid by the respective pre-synaptic signals are highlighted. Scale bar: 50µm, refers to whole panel. (**B, D**) Quantification of the NMJ endplates that are fully innervated, partially denervated or completely denervated at 2 months (**B**) and 1 year of age (**D**) shows no significant changes between control and Dnm2 wt/K562E mice in the number of fully denervated structures at both ages. The number of fully innervated NMJs are mildly reduced in mutants at both ages, and the partially denervated ones show a trend towards an increase in 2 months-old Dnm2 wt/K562E animals, which reaches statistical significance by 1 year of age. Bar heights: Mean; error bars:

s.e.m. (n = 6 control and n = 5 Dnm2 wt/K562E mice (2 months), n = 6 control and n = 8 Dnm2 wt/K562E mice (1 year), at least 100 neuromuscular junctions from each animal were quantified). Two-Way ANOVA with Sidak´s multiple comparisons test. Significance was set at $^{*}p < 0.05$, $^{**}p < 0.01$.

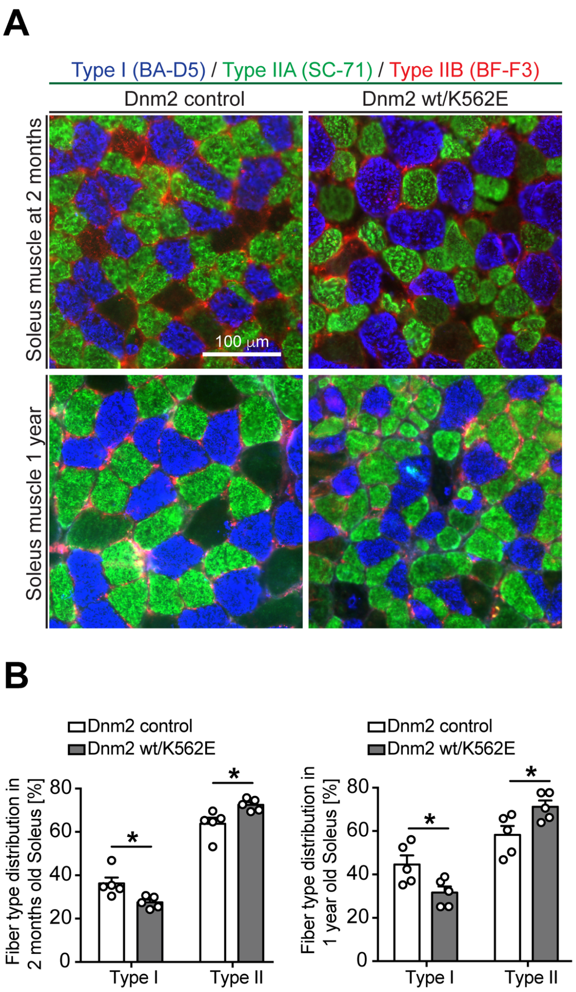

Supplementary Material Figure S6: Checkerboard pattern of fiber-type distribution is preserved in Dnm2 wt/K562E soleus muscle.

(**A**) Exemplary pictures from immunolabelled cryosections of control and Dnm2 wt/K562E soleus muscles derived from 2 months- and 1 year-old mice, probed with isotype-specific antibodies recognizing myosin heavy chains characteristic of type I, or type IIA, or type IIB myofibers. Myofibers not labelled by any of the three antibodies used are likely type IIX. Note that the red signal is also not-specifically present around fibers of all types. The checkerboard random pattern of fiber-type distribution is not disrupted in Dnm2 wt/K562E samples. (**B**) Quantification of the percental abundance of type I and type II (includes the type IIA and IIB) myofibers from control and Dnm2 wt/K562E soleus muscle sections (shown in **A**) reveals only marginal changes in the numbers of both type I and type II fibers between both genotypes. Bar heights: Mean; error bars: s.e.m. (n = 5 mice/genotype at 2 months and 1 year, images from at least 3 sections per mouse were quantified and averaged). Two-Way ANOVA with Sidak´s multiple comparisons test. Significance was set at *$p < 0.05$.

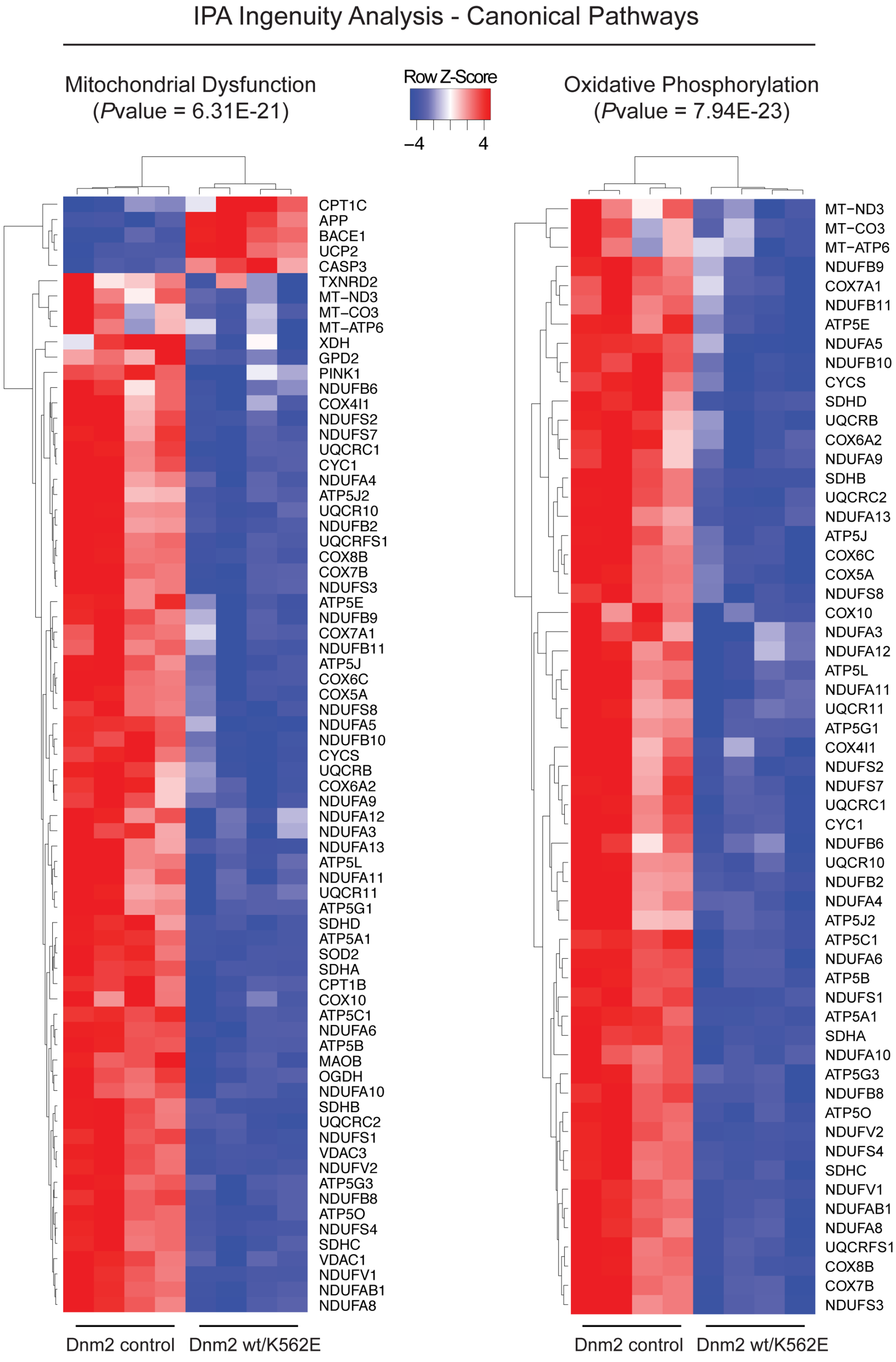

Supplementary Material Figure S7: IPA Ingenuity canonical pathway analysis highlights mitochondrial-related transcripts as differentially regulated in soleus muscles of Dnm2 wt/K562E versus control mice.

IPA Ingenuity analysis of canonical pathways reveals that oxidative phosphorylation and mitochondrial dysfunction are the most significantly represented categories in the dataset (FDR < 0.05 and log2 fold change at least ± 0.58). Heatmap representing the transcripts associated by Ingenuity with each of these 2 canonical pathways confirms they are dominantly downregulated in Dnm2 wt/K562E compared to control soleus muscles (n = 4 mice/genotype).

Figure	Panel	Graph location within panel	Conditions compared directly (condition 1 Vs condition 2)	N condition 1	N condition 2	Mean condition 1	SEM condition 1	Mean condition 2	SEM condition 2	Type of Test	ANOVA F value	ANOVA P value	Ttest (t) value	Degrees of freedom	Multiple comparison test	P Value
Figure 1	C		Control Vs Dnm2 wt/K562E (males) at 2 months	17	14	24.22	0.2349	21.94	0.4095	two-tailed unpaired Student's Ttest			5.031	29		<0.0001
			Control Vs Dnm2 wt/K562E (females) at 2 months	13	15	18.91	0.3119	17.58	0.3327	two-tailed unpaired Student's Ttest			2.889	26		0.0077
	D		Control Vs Dnm2 wt/K562E (males) at 1 year	24	19	34.72	0.6128	27.95	0.5475	two-tailed unpaired Student's Ttest			8.016	41		<0.0001
			Control Vs Dnm2 wt/K562E (females) at 1 year	15	20	29.2	0.8504	25.21	0.7264	two-tailed unpaired Student's Ttest			3.572	33		0.0011
	E		Rotarod, Control Vs Dnm2 wt/K562E at 2 months	10	10	275	17.13	304.8	20.01	two-tailed unpaired Student's Ttest			1.132	18		0.2724
			Grip Strength, Control Vs Dnm2 wt/K562E at 2 months	10	10	4.657	0.3126	4.854	0.2651	two-tailed unpaired Student's Ttest			0.479	18		0.6377
			Hot Plate, Control Vs Dnm2 wt/K562E at 2 months	10	10	15.5	3.597	12.9	1.1	two-tailed unpaired Student's Ttest			0.6912	18		0.4982
			Catwalk BOS, Control Vs Dnm2 wt/K562E at 2 months	10	10	3.281	0.08278	3.241	0.09317	two-tailed unpaired Student's Ttest			0.3227	18		0.7506
			Catwalk, Stride Length, Control Vs Dnm2 wt/K562E at 2 months	10	10	9.354	0.1958	8.566	0.3111	two-tailed unpaired Student's Ttest			2.146	18		0.0457
			Catwalk Duty Cycle, Control Vs Dnm2 wt/K562E at 2 months	10	10	48.1	0.7667	48.13	1.664	two-tailed unpaired Student's Ttest			0.01286	18		0.9899
			Inverted Hang, Control Vs Dnm2 wt/K562E at 2 months	10	10	466.6	56.97	371.8	41.73	two-tailed unpaired Student's Ttest			1.343	18		0.196
			Open Field, Control Vs Dnm2 wt/K562E at 2 months	10	10	355.5	23.44	278.4	10.46	two-tailed unpaired Student's Ttest			3.005	18		0.0076
	F		Rotarod, Control Vs Dnm2 wt/K562E at 1 year	26	28	232.4	20.02	207.1	8.342	two-tailed unpaired Student's Ttest			1.193	52		0.2381
			Grip Strength, Control Vs Dnm2 wt/K562E at 1 year	24	28	3.423	0.2566	3.489	0.1987	two-tailed unpaired Student's Ttest			0.2058	50		0.8378
			Hot Plate, Control Vs Dnm2 wt/K562E at 1 year	12	12	20.17	1.98	19.83	2.191	two-tailed unpaired Student's Ttest			0.1129	22		0.9112
			Catwalk BOS, Control Vs Dnm2 wt/K562E at 1 year	16	16	3.852	0.09104	4.065	0.07254	two-tailed unpaired Student's Ttest			1.831	30		0.077
			Catwalk, Stride Length, Control Vs Dnm2 wt/K562E at 1 year	16	16	9.48	0.3605	8.333	0.2733	two-tailed unpaired Student's Ttest			2.535	30		0.0167
			Catwalk Duty Cycle, Control Vs Dnm2 wt/K562E at 1 year	16	16	55.09	1.441	59.03	1.088	two-tailed unpaired Student's Ttest			2.183	30		0.037
			Open Field, Control Vs Dnm2 wt/K562E at 1 year	15	14	280.4	29.55	179.5	12.25	two-tailed unpaired Student's Ttest			3.074	27		0.0048
	G		mNCV, Control Vs Dnm2 wt/K562E at 1 year	22	22	40.42	2.057	37.69	1.561	two-tailed unpaired Student's Ttest			1.058	42		0.2961
			csNCV, Control Vs Dnm2 wt/K562E at 1 year	22	22	52.17	2.762	52.24	2.639	two-tailed unpaired Student's Ttest			0.01725	42		0.9863
			CMAP, Control Vs Dnm2 wt/K562E at 1 year	22	22	10.41	0.9396	6.238	0.7133	two-tailed unpaired Student's Ttest			3.541	42		0.001
			csAmplitude, Control Vs Dnm2 wt/K562E at 1 year	22	22	38.02	5.053	55.44	7.477	two-tailed unpaired Student's Ttest			1.931	42		0.0603
			Repetitive Stimulation 3 Hz, Control Vs Dnm2 wt/K562E at 1 year	22	22	-0.1591	0.5801	-2.859	0.781	two-tailed unpaired Student's Ttest			2.775	42		0.0082
			Repetitive Stimulation 10 Hz, Control Vs Dnm2 wt/K562E at 1 year	22	22	-1.236	0.6177	-7.332	1.626	two-tailed unpaired Student's Ttest			3.504	42		0.0011
Figure 2	A	left (2 months)	Control Vs Dnm2 wt/K562E	6	5	18.17	3.945	20	3.13	two-tailed unpaired Student's Ttest			0.3528	9		0.7324
		right (1 year)	Control Vs Dnm2 wt/K562E	5	5	17	4.561	16.4	2.227	two-tailed unpaired Student's Ttest			0.1182	8		0.9088
	D		≤2 Control Vs Dnm2 wt/K562E	5	5	0.67371244	0.007278658	0.63063126	0.005094966	Two-Way ANOVA	3.545	0.0144		interaction (4, 40)	Sidak's	< 0.0001
			2 to 3 Control Vs Dnm2 wt/K562E	5	5	0.64127434	0.006360268	0.61129556	0.008079491							0.0063
			3 to 4 Control Vs Dnm2 wt/K562E	5	5	0.64437918	0.006984756	0.62598296	0.002461577							0.1818
			4 to 5 Control Vs Dnm2 wt/K562E	5	5	0.66334678	0.006794657	0.6524784	0.001492622							0.7027
			>5 Control Vs Dnm2 wt/K562E	5	5	0.70552044	0.007127471	0.70401196	0.005836572							>0.9999
	E		Control Vs Dnm2 wt/K562E	5	5	0.6534	0.005875	0.6291	0.004127	two-tailed unpaired Student's Ttest			3.394	8		0.0094
	F		Control Vs Dnm2 wt/K562E	5	5	1522	25.01	1496	32.37	two-tailed unpaired Student's Ttest			0.6357	8		0.5427
	G		≤1 Control Vs Dnm2 wt/K562E	5	5	0.5861768	0.135341228	0.790404	0.130618202	Two-Way ANOVA	2.816	0.0182		interaction (6, 56)	Sidak's	>0.9999
			>1 to 2 Control Vs Dnm2 wt/K562E	5	5	22.836782	0.957989693	24.86521	0.858701708							0.414
			>2 to 3 Control Vs Dnm2 wt/K562E	5	5	34.631734	1.715235015	30.97365	0.479653139							0.0121
			>3 to 4 Control Vs Dnm2 wt/K562E	5	5	27.854332	0.869858991	27.028096	0.566589566							0.9867
			>4 to 5 Control Vs Dnm2 wt/K562E	5	5	11.1030408	1.402089412	12.52529	0.521081193							0.8015
			>5 to 6 Control Vs Dnm2 wt/K562E	5	5	2.7889168	0.513124559	3.5971562	0.440671222							0.9883
			>6 Control Vs Dnm2 wt/K562E	5	5	0.1990188	0.103081492	0.2201948	0.113817587							>0.9999
	H		Control Vs Dnm2 wt/K562E	5	5	2.863	0.05825	2.879	0.03264	two-tailed unpaired Student's Ttest			0.2406	8		0.8159
Figure 3	B		Control Vs P0Cre Dnm2 fl/wt	5	5	1481	56.27	1443	23.13	One-Way ANOVA	59.17	<0.0001		3, 16	Tukey's	0.9164
			Control Vs Dnm2 wt/K562E	5	5	1481	56.27	1472	52							0.9985
			Control Vs P0Cre Dnm2 fl/K562E	5	5	1481	56.27	826.8	22.49							<0.0001
			P0Cre Dnm2 fl/wt Vs Dnm2 wt/K562E	5	5	1443	23.13	1472	52							0.9617
			P0Cre Dnm2 fl/wt Vs P0Cre Dnm2 fl/K562E	5	5	1443	23.13	826.8	22.49							<0.0001
			Dnm2 wt/K562E Vs P0Cre Dnm2 fl/K562E	5	5	1472	52	826.8	22.49							<0.0001
	C		Control Vs P0Cre Dnm2 fl/wt	5	5	0.8	0.3742	0.6	0.2449	One-Way ANOVA	522.2	<0.0001		3, 16	Tukey's	>0.9999
			Control Vs Dnm2 wt/K562E	5	5	0.8	0.3742	0.2	0.2							>0.9999
			Control Vs P0Cre Dnm2 fl/K562E	5	5	0.8	0.3742	536.8	23.46							<0.0001
			P0Cre Dnm2 fl/wt Vs Dnm2 wt/K562E	5	5	0.6	0.2449	0.2	0.2							>0.9999
			P0Cre Dnm2 fl/wt Vs P0Cre Dnm2 fl/K562E	5	5	0.6	0.2449	536.8	23.46							<0.0001
			Dnm2 wt/K562E Vs P0Cre Dnm2 fl/K562E	5	5	0.2	0.2	536.8	23.46							<0.0001
	D		Control Vs P0Cre Dnm2 fl/wt	5	5	1482	56.08	1444	23.16	One-Way ANOVA	1.743	0.1985		3, 16	Tukey's	0.9101
			Control Vs Dnm2 wt/K562E	5	5	1482	56.08	1472	51.99							0.998
			Control Vs P0Cre Dnm2 fl/K562E	5	5	1482	56.08	1364	15							0.2082
			P0Cre Dnm2 fl/wt Vs Dnm2 wt/K562E	5	5	1444	23.16	1472	51.99							0.9609
			P0Cre Dnm2 fl/wt Vs P0Cre Dnm2 fl/K562E	5	5	1444	23.16	1364	15							0.5179
			Dnm2 wt/K562E Vs P0Cre Dnm2 fl/K562E	5	5	1472	51.99	1364	15							0.2726
	F		Control Vs P0Cre Dnm2 fl/wt	5	5	17.2	1.772	14.2	3.056	One-Way ANOVA	1.933	0.1649		3, 16	Tukey's	0.8659
			Control Vs Dnm2 wt/K562E	5	5	17.2	1.772	14.4	3.203							0.8875
			Control Vs P0Cre Dnm2 fl/K562E	5	5	17.2	1.772	22.4	2.731							0.5534
			P0Cre Dnm2 fl/wt Vs Dnm2 wt/K562E	5	5	14.2	3.056	14.4	3.203							>0.9999
			P0Cre Dnm2 fl/wt Vs P0Cre Dnm2 fl/K562E	5	5	14.2	3.056	22.4	2.731							0.1919
			Dnm2 wt/K562E Vs P0Cre Dnm2 fl/K562E	5	5	14.4	3.203	22.4	2.731							0.2085
	G		Control Vs P0Cre Dnm2 fl/wt	5	5	0	0	0	0	One-Way ANOVA	85.45	<0.0001		3, 16	Tukey's	>0.9999
			Control Vs Dnm2 wt/K562E	5	5	0	0	0	0							>0.9999
			Control Vs P0Cre Dnm2 fl/K562E	5	5	0	0	47.8	5.171							<0.0001
			P0Cre Dnm2 fl/wt Vs Dnm2 wt/K562E	5	5	0	0	0	0							>0.9999
			P0Cre Dnm2 fl/wt Vs P0Cre Dnm2 fl/K562E	5	5	0	0	47.8	5.171							<0.0001
			Dnm2 wt/K562E Vs P0Cre Dnm2 fl/K562E	5	5	0	0	47.8	5.171							<0.0001
	I		mNCV, Control Vs P0Cre Dnm2 fl/K562E	5	5	25.5	2.553	4.02	0.4923	two-tailed unpaired Student's Ttest			8.262	8		<0.0001
			csNCV, Control Vs P0Cre Dnm2 fl/K562E	5	5	37.79	6.718	not detected	not detected							
			CMAP, Control Vs P0Cre Dnm2 fl/K562E	5	5	6.44	1.267	1.26	0.1327	two-tailed unpaired Student's Ttest			4.066	8		0.0036
			csAmplitude, Control Vs P0Cre Dnm2 fl/K562E	5	5	36.99	13.56	not detected	not detected							
Figure 4	B	Top (2 months)	0 to 5 Control Vs Dnm2 wt/K562E	5	5	0	0	0	0	Two-Way ANOVA	16.39	<0.0001		interaction (15,128)	Sidak's	>0.9999
			5 to 10 Control Vs Dnm2 wt/K562E	5	5	0	0	0.062893082	0.062893082							>0.9999
			10 to 15 Control Vs Dnm2 wt/K562E	5	5	0.081300814	0.081300814	0.175734748	0.121880692							>0.9999
			15 to 20 Control Vs Dnm2 wt/K562E	5	5	0.229946524	0.15220562	2.352740862	0.692466129							0.9999
			20 to 25 Control Vs Dnm2 wt/K562E	5	5	0.427522304	0.324415313	12.73325015	4.232651124							0.0002
			25 to 30 Control Vs Dnm2 wt/K562E	5	5	4.017328504	1.528513559	29.54472052	4.261911099							<0.0001
			30 to 35 Control Vs Dnm2 wt/K562E	5	5	12.21430002	3.357345799	23.6150546	2.90786283							0.0006
			35 to 40 Control Vs Dnm2 wt/K562E	5	5	31.07725648	3.428855798	14.27437746	2.37539868							<0.0001
			40 to 45 Control Vs Dnm2 wt/K562E	5	5	28.165984	3.660334952	7.607524472	1.69379689							<0.0001
			45 to 50 Control Vs Dnm2 wt/K562E	5	5	15.3476276	3.330132505	4.667098734	1.721623334							0.0018
			50 to 55 Control Vs Dnm2 wt/K562E	5	5	5.78147216	1.906935581	3.414560304	1.692055872							0.9995
			55 to 60 Control Vs Dnm2 wt/K562E	5	5	1.711513066	0.88391967	0.94898705	0.750807235							>0.9999
			60 to 65 Control Vs Dnm2 wt/K562E	5	5	0.445748566	0.340730762	0.285597714	0.226592308							>0.9999
			65 to 70 Control Vs Dnm2 wt/K562E	5	5	0.333333334	0.333333334	0.211640212	0.211640212							>0.9999
			70 to 75 Control Vs Dnm2 wt/K562E	5	5	0	0	0.105820106	0.105820106							>0.9999
			75 to 80 Control Vs Dnm2 wt/K562E	5	5	0.166666666	0.166666666	0	0							>0.9999
		Bottom (1 year)	0 to 5 Control Vs Dnm2 wt/K562E	5	5	0	0	0	0	Two-Way ANOVA	13.56	<0.0001		interaction (15,128)	Sidak's	>0.9999
			5 to 10 Control Vs Dnm2 wt/K562E	5	5	0.238095238	0.238095238	0.379265942	0.292172276							>0.9999
			10 to 15 Control Vs Dnm2 wt/K562E	5	5	0	0	4.060134668	1.713279114							0.9135
			15 to 20 Control Vs Dnm2 wt/K562E	5	5	0.476190476	0.476190476	4.804119194	1.502407494							0.8649
			20 to 25 Control Vs Dnm2 wt/K562E	5	5	0.420183894	0.258261274	9.55175473	1.333155063							0.0183
			25 to 30 Control Vs Dnm2 wt/K562E	5	5	0.238171186	0.145888221	18.17783906	1.961793161							<0.0001
			30 to 35 Control Vs Dnm2 wt/K562E	5	5	2.710884852	0.874963612	20.47056884	3.633172764							<0.0001
			35 to 40 Control Vs Dnm2 wt/K562E	5	5	11.81245844	3.001606414	17.73210936	2.796408055							0.4157
			40 to 45 Control Vs Dnm2 wt/K562E	5	5	22.36602957	4.717516958	12.97178892	1.395477999							0.0134
			45 to 50 Control Vs Dnm2 wt/K562E	5	5	26.06019536	3.758393703	7.11842405	0.913579893							<0.0001
			50 to 55 Control Vs Dnm2 wt/K562E	5	5	15.68683272	1.24614039	2.443357738	0.826524177							<0.0001
			55 to 60 Control Vs Dnm2 wt/K562E	5	5	10.65141608	3.752039305	1.186455954	0.827866873							0.0123
			60 to 65 Control Vs Dnm2 wt/K562E	5	5	5.803644918	4.19367105	0.558958712	0.34326235							0.6185
			65 to 70 Control Vs Dnm2 wt/K562E	5	5	1.985035456	1.720762935	0.545222838	0.398548413							>0.9999
			70 to 75 Control Vs Dnm2 wt/K562E	5	5	1.550861786	1.291138718	0	0							>0.9999
			75 to 80 Control Vs Dnm2 wt/K562E	5	5	0	0	0	0							>0.9999
	D	Top (2 months macrophages)	Control Vs Dnm2 wt/K562E	5	5	63.4	8.452	213.8	19.02	two-tailed unpaired Student's Ttest			7.226	8		<0.0001
		Bottom (1 year macrophages)	Control Vs Dnm2 wt/K562E	5	5	26.95	2.091	181.9	21.32	two-tailed unpaired Student's Ttest			7.232	8		<0.0001
	E	Top (2 months fibers)	Control Vs Dnm2 wt/K562E	5	5	0	0	17.83	5.532	One-sample Ttest			3.223	4		0.0322
		Bottom (1 year fibers)	Control Vs Dnm2 wt/K562E	5	5	0	0	0.4161	0.4161	One-sample Ttest			1	4		0.3739
Figure 6	A	left (males)	Control Vs Dnm2 wt/K562E (males) at 2 months	7	6	44.7	1.26	37.75	1.793	two-tailed unpaired Student's Ttest			3.245	11		0.0078
		left (females)	Control Vs Dnm2 wt/K562E (females) at 2 months	9	10	38.42	1.532	32.78	1.122	two-tailed unpaired Student's Ttest			3.013	17		0.0078
		right (males)	Control Vs Dnm2 wt/K562E (males) at 1 year	13	9	54.73	1.154	37.26	1.302	two-tailed unpaired Student's Ttest			9.927	20		<0.0001
		right (females)	Control Vs Dnm2 wt/K562E (females) at 1 year	5	7	46.4	1.344	30.79	1.279	two-tailed unpaired Student's Ttest			8.236	10		<0.0001
	C		Control Vs Dnm2 wt/K562E	5	5	31.15	3.007	77.73	5.252	two-tailed unpaired Student's Ttest			7.697	8		<0.0001
	D		0 to 5 Control Vs Dnm2 wt/K562E	4	4	0	0	0	0	Two-Way ANOVA	5.223	<0.0001		interaction (17,108)	Sidak's	>0.9999
			5 to 10 Control Vs Dnm2 wt/K562E	4	4	0	0	0	0							>0.9999
			10 to 15 Control Vs Dnm2 wt/K562E	4	4	0	0	0	0							>0.9999
			15 to 20 Control Vs Dnm2 wt/K562E	4	4	0	0	0.14367816	0.14367816							>0.9999
			20 to 25 Control Vs Dnm2 wt/K562E	4	4	0.203252033	0.203252033	2.256442283	0.426281729							>0.9999
			25 to 30 Control Vs Dnm2 wt/K562E	4	4	0.58380903	0.385528831	7.306925098	1.715060001							0.2683
			30 to 35 Control Vs Dnm2 wt/K562E	4	4	2.581845988	1.047942376	13.51458771	3.923124955							0.0027
			35 to 40 Control Vs Dnm2 wt/K562E	4	4	7.240399555	2.862976704	12.69795699	1.684416387							0.6182
			40 to 45 Control Vs Dnm2 wt/K562E	4	4	11.28570869	1.131717172	13.19476318	0.91642603							>0.9999
			45 to 50 Control Vs Dnm2 wt/K562E	4	4	11.46114905	1.522543308	21.6117559	3.754466597							0.0072
			50 to 55 Control Vs Dnm2 wt/K562E	4	4	17.06311793	2.43397391	16.92305308	5.78528381							>0.9999
			55 to 60 Control Vs Dnm2 wt/K562E	4	4	18.6730872	3.125087174	7.194580578	1.66599519							0.0013
			60 to 65 Control Vs Dnm2 wt/K562E	4	4	16.87398875	2.025298128	2.652757325	1.15284597							<0.0001
			65 to 70 Control Vs Dnm2 wt/K562E	4	4	6.785299865	3.378986849	1.705241435	1.161693528							0.7306
			70 to 75 Control Vs Dnm2 wt/K562E	4	4	4.599743245	4.028902748	0.584583133	0.403180938							0.9479
			75 to 80 Control Vs Dnm2 wt/K562E	4	4	2.210002168	1.676988396	0.213675213	0.213675213							>0.9999
			80 to 85 Control Vs Dnm2 wt/K562E	4	4	0.43859649	0.43859649	0	0							>0.9999
			85 to 90 Control Vs Dnm2 wt/K562E	4	4	0	0	0	0							>0.9999
Figure 7	B		Control Vs Dnm2 wt/0	5	5	94	5.236	89.33	3.683	two-tailed unpaired Student's Ttest			0.7289	8		0.4868
	C		0 to 5 Control Vs Dnm2 wt/0	5	5	0	0	0	0	Two-Way ANOVA	0.2745	0.9968		interaction (15, 128)	Sidak's	>0.9999
			5 to 10 Control Vs Dnm2 wt/0	5	5	0	0	0	0							>0.9999
			10 to 15 Control Vs Dnm2 wt/0	5	5	0	0	0.216117218	0.08913309							>0.9999
			15 to 20 Control Vs Dnm2 wt/0	5	5	0.082304526	0.082304526	0.155993432	0.155993432							>0.9999
			20 to 25 Control Vs Dnm2 wt/0	5	5	0.877019562	0.266216313	0.541182186	0.263926785							>0.9999
			25 to 30 Control Vs Dnm2 wt/0	5	5	4.948323252	1.734829593	5.121886832	3.358825352							>0.9999
			30 to 35 Control Vs Dnm2 wt/0	5	5	20.42363982	3.763543233	24.2534595	7.939250841							0.9924
			35 to 40 Control Vs Dnm2 wt/0	5	5	43.4650364	1.975413148	37.93369384	4.895185979							0.8362
			40 to 45 Control Vs Dnm2 wt/0	5	5	25.42114882	4.082979651	26.89663301	6.712539189							>0.9999
			45 to 50 Control Vs Dnm2 wt/0	5	5	4.372234902	1.135776391	4.14829322	1.753214511							>0.9999
			50 to 55 Control Vs Dnm2 wt/0	5	5	0.410292756	0.127332219	0.73274077	0.196234123							>0.9999
			55 to 60 Control Vs Dnm2 wt/0	5	5	0	0	0	0							>0.9999
			60 to 65 Control Vs Dnm2 wt/0	5	5	0	0	0	0							>0.9999
			65 to 70 Control Vs Dnm2 wt/0	5	5	0	0	0	0							>0.9999
			70 to 75 Control Vs Dnm2 wt/0	5	5	0	0	0	0							>0.9999
			75 to 80 Control Vs Dnm2 wt/0	5	5	0	0	0	0							>0.9999
Figure 8	A		CD4+ T Cells, Control Vs Dnm2 wt/K562E	6	6	10.41	0.76605918	11.33166667	0.993755224	two-tailed unpaired Student's Ttest			0.734542	10	Holm-Sidak method	0.926596
			CD8+ T Cells, Control Vs Dnm2 wt/K562E	6	6	8.95	0.427784993	9.111666667	0.550898761	two-tailed unpaired Student's Ttest			0.231784	10	Holm-Sidak method	0.931797
			B Cells, Control Vs Dnm2 wt/K562E	6	6	62.86666667	2.108343215	57.48333333	1.291876327	two-tailed unpaired Student's Ttest			2.17714	10	Holm-Sidak method	0.324533
			Neutrophils, Control Vs Dnm2 wt/K562E	6	6	4.703333333	0.57286221	7.246666667	1.999264309	two-tailed unpaired Student's Ttest			1.22292	10	Holm-Sidak method	0.76174
			Control Vs Dnm2 wt/K562E	6	6	2.106666667	0.101773168	2.691666667	0.260824547	two-tailed unpaired Student's Ttest			2.08946	10	Holm-Sidak method	0.324533
			Macrophages, Control Vs Dnm2 wt/K562E	6	6	2.588333333	0.248279636	2.433333333	0.128443676	two-tailed unpaired Student's Ttest			0.55449	10	Holm-Sidak method	0.931797
			Dendritic cells, Control Vs Dnm2 wt/K562E	6	6	0.073	0.006592926	0.0775	0.00614139	two-tailed unpaired Student's Ttest			0.499435	10	Holm-Sidak method	0.931797
	B		CD4+ T Cells, Control Vs Dnm2 wt/K562E	6	6	8.591666667	0.798045181	9.331666667	0.842490026	two-tailed unpaired Student's Ttest			0.637679	10	Holm-Sidak method	0.888007
			CD8+ T Cells, Control Vs Dnm2 wt/K562E	6	6	6.461666667	0.405416795	6.008333333	0.481202775	two-tailed unpaired Student's Ttest			0.720468	10	Holm-Sidak method	0.888007
			B Cells, Control Vs Dnm2 wt/K562E	6	6	49.85	2.286299776	43.18333333	1.577004897	two-tailed unpaired Student's Ttest			2.4003	10	Holm-Sidak method	0.233613
			Neutrophils, Control Vs Dnm2 wt/K562E	6	6	20.73333333	1.79455968	26.26666667	2.047220338	two-tailed unpaired Student's Ttest			2.03251	10	Holm-Sidak method	0.350973
			Control Vs Dnm2 wt/K562E	6	6	5.215	0.371238917	5.798333333	0.588661854	two-tailed unpaired Student's Ttest			0.838187	10	Holm-Sidak method	0.888007
			Macrophages, Control Vs Dnm2 wt/K562E	6	6	2.971666667	0.286919462	2.38	0.132262366	two-tailed unpaired Student's Ttest			1.87274	10	Holm-Sidak method	0.378021
			Dendritic cells, Control Vs Dnm2 wt/K562E	6	6	0.054666667	0.006711516	0.058	0.008617811	two-tailed unpaired Student's Ttest			0.305168	10	Holm-Sidak method	0.888007
	C		CD4+ T Cells, Control Vs Dnm2 wt/K562E	6	6	13.66666667	0.611918658	15.66666667	0.455826478	two-tailed unpaired Student's Ttest			2.62111	10	Holm-Sidak method	0.143829
			CD8+ T Cells, Control Vs Dnm2 wt/K562E	6	6	8.52	0.186064505	9.323333333	0.301547859	two-tailed unpaired Student's Ttest			2.26718	10	Holm-Sidak method	0.213076
			B Cells, Control Vs Dnm2 wt/K562E	6	6	63.6	0.368781778	58.83333333	0.685403369	two-tailed unpaired Student's Ttest			6.12432	10	Holm-Sidak method	0.000784
			Neutrophils, Control Vs Dnm2 wt/K562E	6	6	3.38	0.375872319	3.865	0.608932125	two-tailed unpaired Student's Ttest			0.6777556	10	Holm-Sidak method	0.884713
			Control Vs Dnm2 wt/K562E	6	6	1.266666667	0.194656392	1.391666667	0.194686357	two-tailed unpaired Student's Ttest			0.454039	10	Holm-Sidak method	0.884713
			Macrophages, Control Vs Dnm2 wt/K562E	6	6	0.623333333	0.034512478	0.64	0.053103672	two-tailed unpaired Student's Ttest			0.263158	10	Holm-Sidak method	0.884713
			Dendritic cells, Control Vs Dnm2 wt/K562E	6	6	0.586666667	0.018012341	0.631666667	0.031135903	two-tailed unpaired Student's Ttest			1.25102	10	Holm-Sidak method	0.66533
	D		CD4+ T Cells, Control Vs Dnm2 wt/K562E	6	6	0.826666667	0.151803967	0.855	0.073970715	two-tailed unpaired Student's Ttest			0.167785	10	Holm-Sidak method	0.969385
			CD8+ T Cells, Control Vs Dnm2 wt/K562E	6	6	1.048333333	0.067202017	1.243333333	0.095382272	two-tailed unpaired Student's Ttest			1.67126	10	Holm-Sidak method	0.553117
			B Cells, Control Vs Dnm2 wt/K562E	6	6	31.33333333	1.79660173	33.18333333	2.680225944	two-tailed unpaired Student's Ttest			0.573347	10	Holm-Sidak method	0.943558
			Neutrophils, Control Vs Dnm2 wt/K562E	6	6	30.43333333	1.800123453	28.28333333	2.605304418	two-tailed unpaired Student's Ttest			0.678938	10	Holm-Sidak method	0.943558
			Control Vs Dnm2 wt/K562E	6	6	9.563333333	0.383646249	8.343333333	0.48049049	two-tailed unpaired Student's Ttest			1.98418	10	Holm-Sidak method	0.422099
			Macrophages, Control Vs Dnm2 wt/K562E	6	6	0.175	0.016482314	0.155	0.016072751	two-tailed unpaired Student's Ttest			0.868744	10	Holm-Sidak method	0.925648
			Dendritic cells, Control Vs Dnm2 wt/K562E	6	6	0.035833333	0.007669927	0.037833333	0.004339099	two-tailed unpaired Student's Ttest			0.226957	10	Holm-Sidak method	0.969385
	E		CD4+ T Cells, Control Vs Dnm2 wt/K562E	6	6	6.29	0.683500792	5.193333333	0.786247063	two-tailed unpaired Student's Ttest			1.05266	10	Holm-Sidak method	0.928919
			CD8+ T Cells, Control Vs Dnm2 wt/K562E	6	6	2.651666667	0.411075149	2.078333333	0.352414686	two-tailed unpaired Student's Ttest			1.05887	10	Holm-Sidak method	0.928919
			B Cells, Control Vs Dnm2 wt/K562E	6	6	46.53333333	3.548489507	44.4	4.464825491	two-tailed unpaired Student's Ttest			0.374059	10	Holm-Sidak method	0.977135
			Neutrophils, Control Vs Dnm2 wt/K562E	6	6	4.233333333	0.749411621	6.283333333	1.98892545	two-tailed unpaired Student's Ttest			0.964512	10	Holm-Sidak method	0.928919
			Control Vs Dnm2 wt/K562E	6	6	8.445000167	2.339774566	8.906666667	1.840789082	two-tailed unpaired Student's Ttest			0.155073	10	Holm-Sidak method	0.985564
			Macrophages, Control Vs Dnm2 wt/K562E	6	6	19.47666667	3.721627482	18.91333333	4.010239948	two-tailed unpaired Student's Ttest			0.102966	10	Holm-Sidak method	0.985564
			Dendritic cells, Control Vs Dnm2 wt/K562E	6	6	2.276666667	0.152745249	2.551666667	0.289049208	two-tailed unpaired Student's Ttest			0.841169	10	Holm-Sidak method	0.928919
Supplementary Figure 1			Control Vs Dnm2 wt/K562E	3	3	14.88	0.2599	15.38	0.119	two-tailed unpaired Student's Ttest			1.738	4		0.1572
Supplementary Figure 2	C		≤2 Control Vs Dnm2 wt/K562E	6	5	0.675492133	0.014795969	0.67126442	0.011237742	Two-Way ANOVA	0.2104	0.8885		interaction (3, 35)	Sidak's	0.9958
			2 to 3 Control Vs Dnm2 wt/K562E	6	5	0.651808083	0.008902846	0.63821606	0.00765009							0.7612
			3 to 4 Control Vs Dnm2 wt/K562E	6	5	0.670493467	0.007079593	0.651947	0.004408542							0.5042
			>4 Control Vs Dnm2 wt/K562E	6	4	0.725681833	0.006708222	0.7133119	0.00310737							0.8486
	D		Control Vs Dnm2 wt/K562E	6	5	0.6636	0.009391	0.6501	0.006776	two-tailed unpaired Student's Ttest			1.127	9		0.2891
	E		Control Vs Dnm2 wt/K562E	6	5	1542	84.75	1513	36.82	two-tailed unpaired Student's Ttest			0.2887	9		0.7794
	F		≤1 Control Vs Dnm2 wt/K562E	6	5	1.253833167	0.426110659	0.9600632	0.097059406	Two-Way ANOVA	0.03435	0.9998		interaction (6, 63)	Sidak's	>0.9999
			1 to 2 Control Vs Dnm2 wt/K562E	6	5	30.66364333	2.943476432	30.426932	1.406494931							>0.9999
			2 to 3 Control Vs Dnm2 wt/K562E	6	5	48.71802167	1.152911797	49.43269	1.147558412							0.9998
			3 to 4 Control Vs Dnm2 wt/K562E	6	5	17.236196	2.209266332	17.258612	1.300508355							>0.9999
			4 to 5 Control Vs Dnm2 wt/K562E	6	5	2.030311233	0.543885207	1.8956716	0.432108768							>0.9999
			5 to 6 Control Vs Dnm2 wt/K562E	6	5	0.097994833	0.066555983	0.0260296	0.015963842							>0.9999
			>6 Control Vs Dnm2 wt/K562E	6	5	0	0	0	0							>0.9999
	G		Control Vs Dnm2 wt/K562E	6	5	2.383	0.0742	2.387	0.03761	two-tailed unpaired Student's Ttest			0.04641	9		0.964
Supplementary Figure 3	B		Control Vs Dnm2 wt/K562E	7	5	994	31.36	989	31.32	One-Way ANOVA	0.03091	0.9696		2,14	Tukey's	0.9925
			Control Vs P0Cre Dnm2 fl/K562E	7	5	994	31.36	983.4	26.06							0.9667
			Dnm2 wt/K562E Vs P0Cre Dnm2 fl/K562E	5	5	989	31.32	983.4	26.06							0.9919
	C		Control Vs Dnm2 wt/K562E	7	5	212.4	18.16	197	11.65	One-Way ANOVA	0.3132	0.7361		2,14	Tukey's	0.7865
			Control Vs P0Cre Dnm2 fl/K562E	7	5	212.4	18.16	197	16.34							0.7865
			Dnm2 wt/K562E Vs P0Cre Dnm2 fl/K562E	5	5	197	11.65	197	16.34							>0.9999
	D		Control Vs Dnm2 wt/K562E	7	5	1206	24.06	1186	23.67	One-Way ANOVA	0.4038	0.6753		2,14	Tukey's	0.7933
			Control Vs P0Cre Dnm2 fl/K562E	7	5	1206	24.06	1180	14.75							0.6895
			Dnm2 wt/K562E Vs P0Cre Dnm2 fl/K562E	5	5	1186	23.67	1180	14.75							0.985
	E		Control Vs Dnm2 wt/K562E	7	5	14.57	1.172	11.8	1.393	One-Way ANOVA	1.098	0.3605		2,14	Tukey's	0.5222
			Control Vs P0Cre Dnm2 fl/K562E	7	5	14.57	1.172	11.2	2.8							0.3905
			Dnm2 wt/K562E Vs P0Cre Dnm2 fl/K562E	5	5	11.8	1.393	11.2	2.8							0.973
	G		Control Vs Dnm2 wt/K562E	7	5	2539	83.89	2676	232.1	One-Way ANOVA	1.218	0.3252		2,14	Tukey's	0.7715
			Control Vs P0Cre Dnm2 fl/K562E	7	5	2539	83.89	2344	108.6							0.599
			Dnm2 wt/K562E Vs P0Cre Dnm2 fl/K562E	5	5	2676	232.1	2344	108.6							0.2982
	H		Control Vs Dnm2 wt/K562E	7	5	644.3	42.32	605.4	45.58	One-Way ANOVA	1.912	0.1844		2,14	Tukey's	0.7896
			Control Vs P0Cre Dnm2 fl/K562E	7	5	644.3	42.32	726.6	35.16							0.3683
			Dnm2 wt/K562E Vs P0Cre Dnm2 fl/K562E	5	5	605.4	45.58	726.6	35.16							0.1736
	I		Control Vs Dnm2 wt/K562E	7	5	3183	53.34	3282	222.2	One-Way ANOVA	0.6365	0.5438		2,14	Tukey's	0.8391
			Control Vs P0Cre Dnm2 fl/K562E	7	5	3183	53.34	3070	78.62							0.7956
			Dnm2 wt/K562E Vs P0Cre Dnm2 fl/K562E	5	5	3282	222.2	3070	78.62							0.5138
	J		Control Vs Dnm2 wt/K562E	7	5	40.29	6.647	26.4	1.6	One-Way ANOVA	3.673	0.0522		2,14	Tukey's	0.1851
			Control Vs P0Cre Dnm2 fl/K562E	7	5	40.29	6.647	21.2	4.212							0.0551
			Dnm2 wt/K562E Vs P0Cre Dnm2 fl/K562E	5	5	26.4	1.6	21.2	4.212							0.7971
	K		P0Cre Control* Vs P0Cre Dnm2 wt/K562E*	5	5	1	0.0248	1.042	0.04217	One-Way ANOVA	0.9213	0.4244		2,12	Tukey's	0.8688
			P0Cre Control* Vs P0Cre Dnm2 fl/K562E*	5	5	1	0.0248	0.9313	0.0879							0.6887
			P0Cre Dnm2 wt/K562E* Vs P0Cre Dnm2 fl/K562E*	5	5	1.042	0.04217	0.9313	0.0879							0.3991
Supplementary Figure 4	C		Control Vs Dnm2 wt/K562E	5	5	0.07597	0.07597	10.2	3.142	two-tailed unpaired Student's Ttest			3.222	8		0.0122
	D	left side	Control Vs Dnm2 wt/K562E (males) at 2 months	14	12	9.482	0.2856	7.625	0.326	two-tailed unpaired Student's Ttest			4.304	24		0.0002
			Control Vs Dnm2 wt/K562E (females) at 2 months	9	10	7.167	0.5115	6.82	0.2855	two-tailed unpaired Student's Ttest			0.608	17		0.5512
		right side	Control Vs Dnm2 wt/K562E (males) at 1 year	13	9	11.23	0.4249	7.878	0.3475	two-tailed unpaired Student's Ttest			5.696	20		<0.0001
			Control Vs Dnm2 wt/K562E (females) at 1 year	5	7	9.02	0.3597	6.329	0.4628	two-tailed unpaired Student's Ttest			4.271	10		0.0016
	E		Control Vs Dnm2 wt/K562E	5	5	4.305	1.262	5.537	0.859	two-tailed unpaired Student's Ttest			0.807	8		0.443
Supplementary Figure 5	B		Fully innervated	6	5	93.049	1.292	85.703	3.52	Two-Way ANOVA	5.624	0.0091		interaction (2, 27)	Sidak's	0.0331
			Partially denervated	6	5	6.405	1.256	10.707	2.803							0.3235
			Completely denervated	6	5	0.545	0.258	3.589	1.242							0.609
	D		Fully innervated	6	8	79.177	3.708	58.607	5.661	Two-Way ANOVA	11.16	0.0002		interaction (2, 36)	Sidak's	0.0019
			Partially denervated	6	8	18.355	3.188	33.136	2.97							0.0321
			Completely denervated	6	8	2.468	1.064	8.257	3.514							0.6562
Supplementary Figure 6	B	left side	2 months Fibertype I Control Vs Dnm2 wt/K562E	5	5	36.213	2.783	27.519	1.243	Two-Way ANOVA	16.27	0.001		interaction (1, 16)	Sidak's	0.0229
			2 months Fibertype II Control Vs Dnm2 wt/K562E	5	5	63.787	2.783	72.481	1.243							0.0229
		right side	1 year Fibertype I Control Vs Dnm2 wt/K562E	5	5	43.398	4.027	30.754	2.761	Two-Way ANOVA	13.41	0.0021		interaction (1, 16)	Sidak's	0.0391
			1 year Fibertype II Control Vs Dnm2 wt/K562E	5	5	56.602	4.027	69.246	2.761							0.0391

Supplementary Material Figure S8: Statistics Summary
